# Epithelial plasticity in COPD results in cellular unjamming due to an increase in polymerized actin

**DOI:** 10.1242/jcs.258513

**Published:** 2022-02-24

**Authors:** Baishakhi Ghosh, Kristine Nishida, Lakshmana Chandrala, Saborny Mahmud, Shreeti Thapa, Carter Swaby, Si Chen, Atulya Aman Khosla, Joseph Katz, Venkataramana K. Sidhaye

**Affiliations:** 1 Department of Environmental Health and Engineering, Bloomberg School of Public Health, Baltimore, Maryland, 21205, USA; 2 Department of Pulmonary and Critical Care Medicine, Johns Hopkins School of Medicine, Johns Hopkins University, Baltimore, Maryland, 21224, USA; 3 Department of Mechanical Engineering, Johns Hopkins Whiting School of Engineering, Johns Hopkins University, Baltimore, Maryland, 21218, USA; 4 Department of Chemical and Biomolecular Engineering, Johns Hopkins Whiting School of Engineering, Johns Hopkins University, Baltimore, Maryland, 21218, USA

**Keywords:** COPD, Cellular unjamming, Cofilin-1, Epithelial plasticity, Polymerized actin, pEMT

## Abstract

The airway epithelium is subjected to insults such as cigarette smoke (CS), a primary cause of chronic obstructive pulmonary disease (COPD) and serves as an excellent model to study cell plasticity. Here, we show that both CS-exposed and COPD-patient derived epithelia (CHBE) display quantitative evidence of cellular plasticity, with loss of specialized apical features and a transcriptional profile suggestive of partial epithelial-to-mesenchymal transition (pEMT), albeit with distinct cell motion indicative of cellular unjamming. These injured/diseased cells have an increased fraction of polymerized actin, due to loss of the actin-severing protein cofilin-1. We observed that decreasing polymerized actin restores the jammed state in both CHBE and CS-exposed epithelia, indicating that the fraction of polymerized actin is critical in unjamming the epithelia. Our kinetic energy spectral analysis suggests that loss of cofilin-1 results in unjamming, similar to that seen with both CS exposure and in CHBE cells. The findings suggest that in response to chronic injury, although epithelial cells display evidence of pEMT, their movement is more consistent with cellular unjamming. Inhibitors of actin polymerization rectify the unjamming features of the monolayer.

This article has an associated First Person interview with the first author of the paper.

## Abstract (precis)

**Summary:** COPD-patient-derived epithelia show quantifiable plasticity that is replicated by repetitive *in vitro* smoke exposure and is caused by increased actin polymer mass and loss of cofilin-1.

## INTRODUCTION

‘Cellular plasticity’ is generally defined as the ability of a cell to alter its structure and shape to adapt to local cues, often resulting in altered cellular identity and function along a phenotypic spectrum (Yuan et al., 2019). Plasticity has been extensively studied in neurologic diseases and cancer, with the understanding that the structural and functional changes can increase adaptability but may disrupt cellular homeostasis and result in disease (Kolb and Gibb, 2011; Yuan et al., 2019; Surcel et al., 2019). Epithelial cells, which are arranged as cohesive sheets, line body surfaces for specialized roles in absorption, secretion or barrier function. The airway epithelia act as a physical barrier between the external and internal environment and adapt to exposure of more than 10,000 l of air daily. A normal pseudostratified airway epithelium undergoes cellular proliferation and differentiation to serve critical roles in monolayer barrier integrity, ciliary beat frequency (CBF) and polarity, all of which are required to maintain tissue homeostasis. However, additional pollutants present in the airstream can damage the epithelium, causing the airway epithelium to serve as an ideal model of chronic injury response.

Chronic obstructive pulmonary disease (COPD) is characterized by a progressive decline in lung function that can occur even after the injurious agent, often tobacco smoke, is removed. It is on target to be the third leading cause of death worldwide and is the fourth leading cause of mortality in the USA (Lozano et al., 2012; Mannino and Buist, 2007). Although dysfunction of several pro-inflammatory pathways have been implicated in COPD, targeting these do not abrogate or reverse disease. Given the societal impact of this disease and the lack of disease-modifying therapeutics, we are in desperate need of new strategies.

Here, we quantify the phenotypic changes that occur in COPD-patient-derived diseased and cigarette smoke (CS)-injured airway epithelial cells. We have identified measures of tissue integrity for healthy airway epithelium, namely monolayer permeability, polarity and height and expression of cell-cell adhesion proteins, all of which constitute the mechanical barrier (Welsh, 2017; Ganesan et al., 2013). We have previously shown that repetitive CS exposure disrupts this integrity (Nishida et al., 2017; Aggarwal et al., 2013), and confirmed in our model that persistent exposure to CS results in epithelial remodeling with loss of cilia (Livraghi and Randell, 2007; Park et al., 2006) and a decrease in CBF (Chandrala et al., 2019), to alter mucociliary clearance (Thomas et al., 2008; Yaghi et al., 2012; Thomas et al., 2010). As a comparison, we also studied epithelial remodeling where cells lose their epithelial identity, as occurs in epithelial-to-mesenchymal transition (EMT). In EMT, there is a complete loss of cell–cell adhesion and apical–basal polarity, with increased cellular motility, although partial EMT (pEMT) may occur with intermediate phenotypes (Thiery, 2003; Mitchel et al., 2020). Although mesenchymal cells migrate as single cells solitary, epithelial plasticity can encompass other patterns of cellular movement as well, including collective motion (Huang et al., 2015) resulting from what has been termed the unjamming transition (UJT) (Mitchel et al., 2020; Park et al., 2016; O'Sullivan et al., 2020; Lin et al., 2021; Kim et al., 2020, 2013). pEMT and cellular unjamming may both co-exist in response to stimuli (Mitchel et al., 2020; O'Sullivan et al., 2020). However, analysis of the plasticity that occurs in COPD epithelial cells remains limited.

By quantifying the changes that occur from exposure to CS and comparing these to cells derived from patients with COPD (COPD human bronchial epithelia; CHBE), we demonstrate here that there is a co-existence of pEMT and cellular unjamming, with expression of transcriptional markers of pEMT. We also observed that the cellular movement and the corresponding cellular energy cascades in CHBE and CS are indicative of cellular unjamming with collective motion (UJT). Spectral analysis was used to characterize the size, spatial distributions and energy of cell motions within the different cultures. This enabled us to highlight similarities and discrepancies in the eddy sizes and strength. Furthermore, the spectral slopes were compared to those of the kinetic energy of eddies and scalars in typical planar turbulent flows (Kraichnan, 1971), as well as to recently measured and simulated spectra of motile cell cultures (Lin et al., 2021). Our data indicate that cellular unjamming results from an increase in the polymerized fraction of actin and decreasing the level of polymerized actin improves monolayer integrity and restores cellular jamming. Both the CS-injured and COPD cells express less cofilin-1, an actin-severing protein, and restoring cofilin-1 to normal levels induces cellular jamming and improves monolayer integrity, but overexpression of cofilin-1 increases mesenchymal-type movement.

## RESULTS

### Evidence for epithelial plasticity in CHBE and CS-exposed non-diseased epithelia

**Fig. 1.**
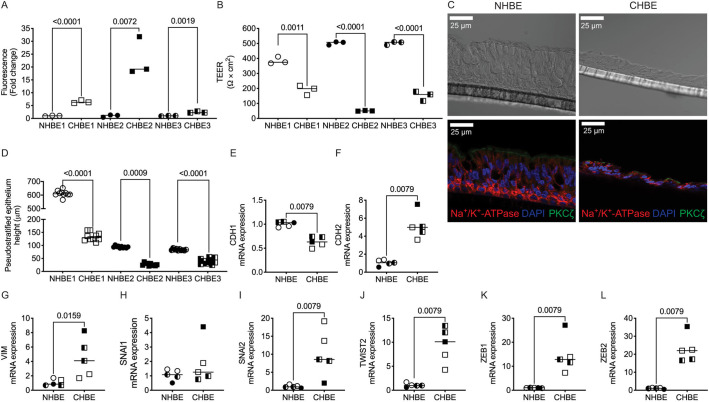
**COPD epithelia demonstrate cellular plasticity with worsened monolayer barrier integrity, lower monolayer height and increased expression of mesenchymal markers.** (A) FITC–dextran permeability is greater in gender- and age-matched COPD epithelial samples (CHBE) than NHBE. (B) Transepithelial resistance (TEER) is lower in gender and age-matched CHBE than NHBE. (C) Immunofluorescence staining of apical polarity (PKC-ζ, green) and basal polarity (Na^+^/K^+^-ATPase, red) shows preservation of polarity in both non-diseased epithelia (NHBE) and CHBE, although the CHBE has a much shorter monolayer height. Images representative of three donors per group (two inserts per donor). (D) The height of pseudostratified epithelium of the NHBE is greater than that of CHBE. (E­–L) There is decreased mRNA expression of epithelial (CDH1) in CHBE compared to age- and gender-matched NHBE (E), and an increase in mRNA expression of mesenchymal markers (CDH2, VIM, SNAl1, SNAl2, TWIST2, ZEB1 and ZEB2) relative to GAPDH in CHBE as analyzed by qPCR (F–L). Data is generated from three donors per group (age- and gender-matched and one to three inserts per donor). Data is expressed with median bars. Statistics determined by Mann–Whitney test, with *P*<0.05 considered statistically significant.

Although grown under identical conditions, the pseudostratified age-matched CHBE monolayer is leakier than non-diseased human bronchial epithelia (NHBE), with a tenfold increase in FITC–dextran flux (Fig. 1A) and 50% lower transepithelial electrical resistance (TEER) (Fig. 1B) (Nishida et al., 2017). The CHBE monolayer was disorganized compared to NHBE, albeit with evidence of apical-basal polarization with a significant number of cilia present and a polarized distribution of protein kinase C (PKC)-ζ and the Na^+^/K^+^-ATPase, despite having a shorter monolayer height (Fig. 1C,D).

Compared to NHBE, the mRNA expression of *CDH1* was significantly lower in CHBE cells (Fig. 1E). In contrast, the mRNA expression of other EMT markers, such as *CDH2*, *VIM*, *SNAI1*, *SNAI2*, *TWIST2*, *ZEB1* and *ZEB2*, was higher in CHBE suggestive of a mesenchymal transcriptional program (Fig. 1F–L). In CHBE cells, the percentage of ciliated cells (percentage of pixels moving) was reduced by 60%, and cells had a lower CBF, albeit with significant donor-specific variability (Fig. S1A,B). To determine whether the plasticity indicated a transition to a mesenchymal cell or epithelial metaplasia, we looked for epithelial markers suggestive of squamous metaplasia. In fact, we found that ∼8.5-fold higher levels of p53 in CHBE as compared to NHBE, and although there was a trend towards an increase in involcurin (*IVL*), this was not found to be statistically significant, due to donor variability (Fig. S1C,D). Also, as we have demonstrated previously (Nishida et al., 2017), the protein expression of E-cadherin was significantly decreased in CHBE as compared to NHBE. The quantitative changes in the phenotype and transcriptional changes suggest evidence of *in vitro* persistence of epithelial plasticity in CHBE, with suggestion of mesenchymal transition.

**Fig. 2.**
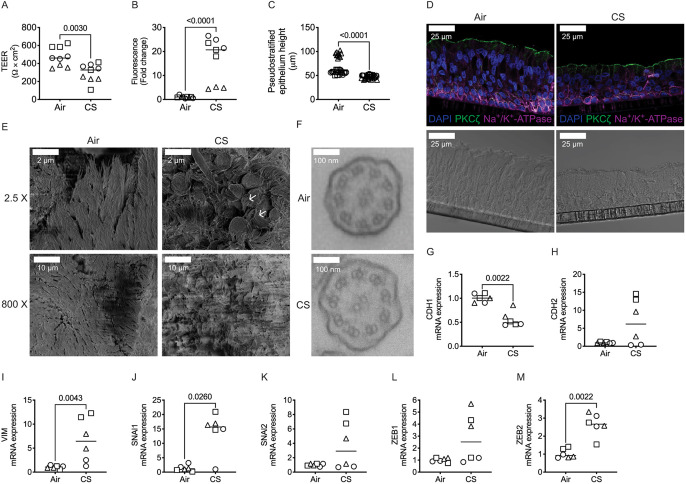
**Repetitive exposure to cigarette smoke drives epithelial plasticity with worsened monolayer barrier integrity, lower monolayer height and increased expression of mesenchymal markers.** (A,B) In CS-exposed NHBE cells TEER is lower (A), and FITC–dextran permeability is greater (B) than in NHBE cells exposed to air. (C) CS exposure in NHBE cells results in a quantitative decrease in the height of pseudostratified epithelium as compared to air-exposed NHBE. (D) Immunofluorescence staining shows preservation of apical-basal polarity (apical, PKC-ζ, green; basolateral, Na^+^/K^+^-ATPase, magenta) taken with a 40× oil objective. Scale bar: 25 µm. (E) Scanning electron microscopy shows preservation of specialized apical structures such as cilia (images taken at 2.5× and 800×). Scale bars: 2 µm, 10 µm. (F) Transmission electron microscopy images showing preservation of the 9+2 ciliary structure in despite CS exposure. Images in D–F representative of three donors per group (two inserts per donor). (G–M) Basal mRNA expression of the epithelial marker *CDH1* is decreased with CS exposure (G), and mesenchymal markers (*CDH2*, *VIM*, *SNAI1*, *SNAI2*, *ZEB1* and *ZEB2*) relative to GAPDH is increased (H–M) with CS exposure than in NHBE cells exposed to air as analyzed by qPCR. Data is generated from three donors (two to three inserts per donor) with graphs showing median bars. Statistics determined by Mann–Whitney test, with *P*<0.05 considered statistically significant.

To determine whether CS exposure similarly altered the epithelial phenotype, we quantified epithelial plasticity after 10 days of CS exposure. The CS-exposed NHBE were leakier, with a 40% decrease in TEER and a tenfold increase in FITC–dextran permeability (Fig. 2A,B), a reduction in CBF by 18% and a 40% loss of cilia (as measured by pixels moving) (Fig. S1E,F) relative to air control. Repetitive exposure to CS also caused a reduction in the height of the pseudostratified monolayer, and an increase in mesenchymal markers, but preserved epithelial polarity and persistence of cilia, as compared to air-exposed NHBE, indicating that the cells retain an epithelial phenotype, quantitatively approaching the changes in CHBE cells (Fig. 2C,D). Scanning electron micrography (SEM) of CS-exposed epithelia showed curling of cilia compared to that in the air control; however, there was no difference in 9+2 arrangement of microtubules as seen by transmission electron micrography (TEM) (Fig. 2E,F). We also observed that repetitive exposure to CS significantly lowered mRNA expression of *CDH1* (Fig. 2G), and there was an increased expression of EMT (Fig. 2H–M) and squamous cell metaplasia markers compared to the air-exposed epithelia (Fig. S1G,H).

### Effect of EMT inducer supplement on the barrier function of the primary bronchial epithelia

**Fig. 3.**
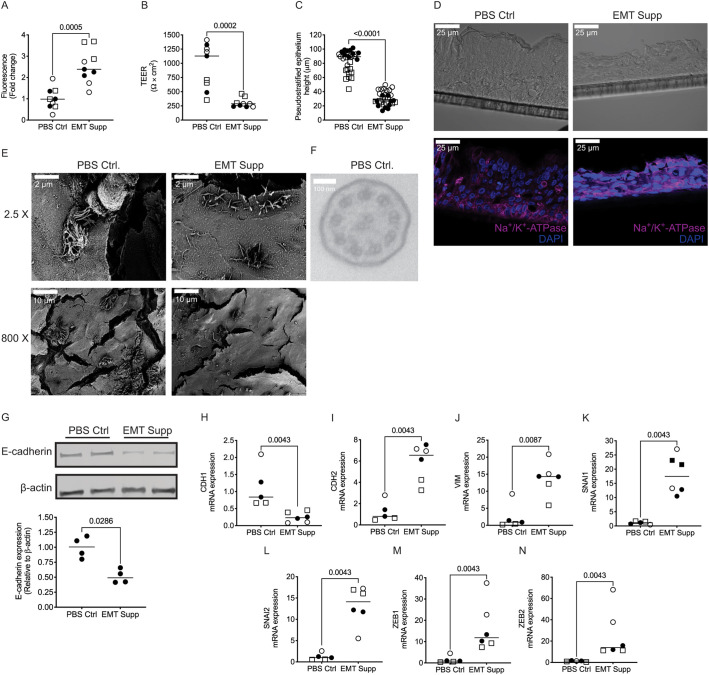
**Comparative analysis of epithleial plasticity of pEMT in primary non-diseased human bronchial epithelia.** (A) NHBE cells undergoing pEMT induced by treatment with 2× EMT inducer supplement (EMT supp) display increased FITC–dextran permeability compared to control PBS-treated (PBS Ctrl) cells. (B) NHBE cells undergoing pEMT display decreased TEER. (C) NHBE cells undergoing pEMT have a lower height of pseudostratified epithelium. (D) There is an expected loss of apical-basal polarity in the cells undergoing EMT as determined by immunofluorescence (basolateral, Na^+^/K^+^-ATPase, magenta). Taken with a 40× oil objective. Scale bars: 25 µm. (E) There is a significant loss of specialized apical structures in cells undergoing pEMT such as cilia, as assessed using scanning electron microscopy. Images taken at 2.5× and 800×. Scale bars: 2 µm, 10 µm. (F) Using transmission electron microscopy, cilia were identified in NHBE treated with PBS Ctrl (top panel); however, there were no cilia evident to capture in the EMT sample, highlighting the loss of specialized apical structures. Images in D–F representative of three donors per group (two inserts per donor). (G) Representative blots (top panel) and quantification (bottom panel) showing decreased E-cadherin expression in cells treated with EMT Supp compared to those treated with PBS Ctrl as detected by western blotting. β-actin levels are shown as a loading control. Blot representative of four inserts from one donor. (H–N) Basal mRNA expression of the epithelial marker *CDH1* is lower in pEMT-induced NHBE cells (H), and levels of mesenchymal markers (*CDH2*, *VIM*, *SNAI1*, *SNAI2*, *ZEB1* and *ZEB2*) relative to GAPDH are increased (I–N) with treatment with EMT Supp were compared to PBS Ctrl, as analyzed by qPCR. Data is generated from three donors (two to four inserts per donor). Data is expressed with median bars. Statistics determined by Mann–Whitney test, with *P*<0.05 considered statistically significant.

To determine whether the plasticity seen in CHBE and NHBE with CS was phenotypically similar to that seen upon EMT, we induced EMT in a confluent pseudostratified epithelial monolayer from non-diseased epithelia by treating them with 2× EMT-inducer supplement (EMT supplement, R&D Systems). This treatment induced pEMT, with a ∼50% leakier and shorter monolayer (Fig. 3A–D). Furthermore, the epithelia lost cell polarity, with altered localization of the basal marker Na^+^/K^+^-ATPase (Fig. 3D) and loss of apical cilia (Fig. S1I). The decrease in ciliary number was confirmed by SEM (Fig. 3E), and with too few cilia to capture the cross-section of the cilia by TEM to confirm the normal 9+2 microtubule arrangement, the existing cilia maintained a similar CBF to that in normal cells (Fig. 3F; Fig. S1J).

After undergoing pEMT, the expression of E-cadherin decreased compared to PBS control-treated (Fig. 3G). Also, the mRNA expression of CDH1 was lower (Fig. 3H), and there was higher expression of both mesenchymal markers (Fig. 3I–N) and squamous cell metaplasia markers in the pEMT cells (Fig. S1K,L).

### Unjamming effect of CS

**Fig. 4.**
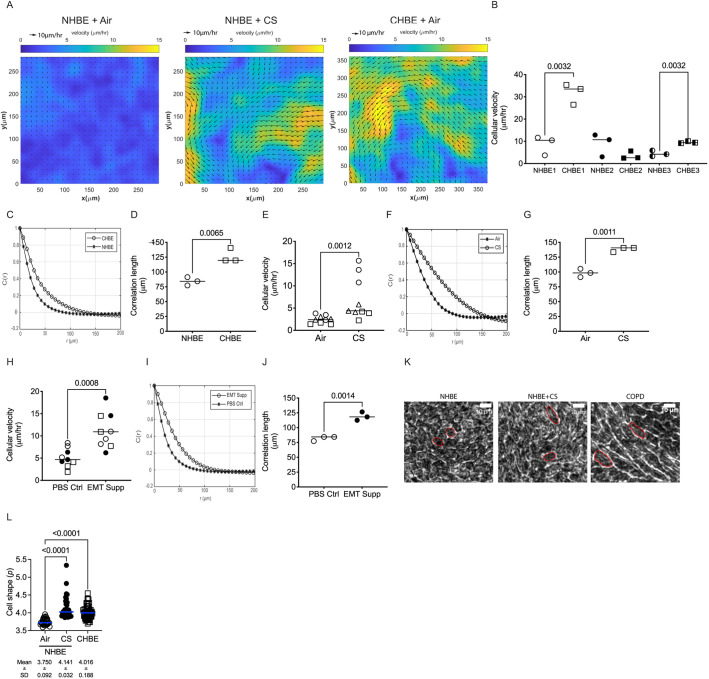
**Epithelial plasticity is associated with increased cell velocity.** (A) Representative heat maps of cell velocity of NHBE exposed to air or cigarette smoke (CS) and CHBE exposed to clean air for 10 days, showing increase in velocity in injured and diseased cells. (B–D) CHBE typically have increased mean cell velocity (B) with CHBE having an altered distribution of spatial autocorrelation function (C) and have a higher correlation length (D) as compared to age- and gender-matched NHBE epithelia. (E–G) CS-exposed NHBE have a higher mean cell velocity (E) with an altered distribution of spatial autocorrelation function (F) and increases in the correlation length (G) in air-exposed NHBE. (H–J) Cells undergoing pEMT induced by treatment with EMT inducer supplement (EMT supp) show increased mean cell velocity (H) with an altered distribution of the spatial autocorrelation function (I) and increased correlation length (J) compared to age- and gender-matched NHBE treated with PBS Ctrl. (K) Representative images of three donors (three inserts per donor) used for cell shape factor quantification in NHBE exposed to air or CS and CHBE. (L) Comparison of quantified cell shape among NHBE exposed to air or CS and CHBE demonstrated an increased cell shape factor in CS-exposed and CHBE cells. Data is generated from three donors (three inserts per donor) and the mean±s.d. is given underneath in L. Graphs are generated with median bars for cell velocity and correlation length. Statistics determined by Mann–Whitney test (B,D,E,G,H,J) or Kruskal–Wallis test followed by Dunn's multiple comparison test (L), with *P*<0.05 considered statistically significant.

Given the transcriptional suggestion of pEMT, we sought to determine whether cellular plasticity in the diseased (CHBE) and injured (CS-exposed) epithelial cells increased cellular motility. We found that the disrupted monolayer and epithelial disorganization seen in CHBE were associated with either a delay in the formation of a jammed-like state or, in some patients, a complete lack of jamming. Similarly, normal epithelium unjammed after CS exposure (Fig. 4A,B). This is consistent with, but much more severe than, what has been observed in asthmatic epithelium (Park et al., 2015). To further elaborate on the dynamics of cooperative cellular mobility, we calculated the spatial autocorrelation function C(r) (Park et al., 2015), from which we estimated the correlation length. The negative values of the correlation function are indicative of swirl patterns in the cell motion, and the correlation length represents a distance over which the cell motion was collective. The correlation length of CHBE was greater than that seen with NHBE (Fig. 4C,D), indicating larger intercellular coordination of movement with visualized swirling motions (Movies 1, 2). Normal cells with a confluent monolayer and minimal cell movement transitioned to an unjammed state with CS exposure (Fig. 4E; Movies 1 and 3), again with a corresponding increase in correlation length (Fig. 4F,G), indicating that the cells are moving together in a coordinated manner. These data indicate that the insult could cause the cells to transition to a collective movement, which is termed UJT (Garcia et al., 2015; Park et al., 2016; Mitchel et al., 2020), resulting in a fluid-like behavior, even in the absence of changes in cell density, thereby implicating a change in cell shape. As a comparison, the cells that underwent pEMT also had an increase in cell movement (Fig. 4H–J).

With CS exposure and in CHBE, we found an elongation of cells, as has been found to occur previously upon unjamming (O'Sullivan et al., 2020; Mitchel et al., 2020), by manually tracing randomly selected cells as depicted in Fig. 4K. We calculated the cell shape factor as described previously (Park et al., 2015), and found that there was an increase in the CHBE and CS exposed NHBE cell shape factor to levels that would be predicted to cause unjamming (Fig. 4L).

### Unjamming of epithelial cells is associated with increase in polymerized actin

**Fig. 5.**
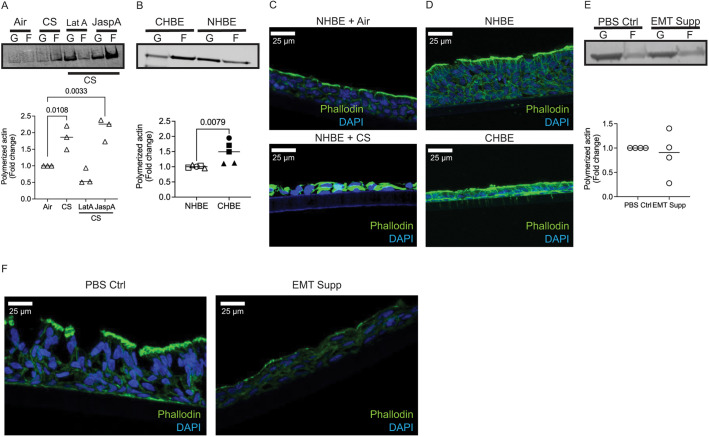
**Both cigarette smoke exposed non-diseased and COPD-derived epithelia have an increased fraction of polymerized actin.** (A) Representative western blot of globular (G) actin and filamentous (F) actin (top panel) and the quantification of polymerized actin (bottom panel) demonstrates that there is a higher polymerized fraction of actin in CS-exposed cells and in cells treated with the polymerizing agent (JaspA), while treatment with actin depolymerizing agent (LatA) reduces the fraction of polymerized actin. Data is representative of three inserts from one donor. (B) Representative western blot of G and F actin (top panel) and quantification of polymerized actin (bottom panel) in age and gender matched cells demonstrate an increase in polymerized fraction of actin in CHBE. Data is representative of three donors (one to two inserts per donor). (C,D) Representative image of immunofluorescence demonstrates increased fluorescence of phalloidin in (C) NHBE exposed to CS and (D) CHBE compared to their respective controls. Data is representative of two donors (two inserts per donor) with images taken under identical exposures. Scale bars: 25 µm. (E) Cells undergoing pEMT do not have an increased fraction of polymerized actin, as shown by the representative western blot of G-actin and F-actin (top panel) and quantification of polymerized actin (bottom panel) of in NHBE treated with PBS Ctrl and EMT Supp (2× EMT inducer supplement). Data is representative of four inserts from one donor. (F) A decrease in phalloidin is seen in sections of cells undergoing pEMT (representative image of immunofluorescence staining of phalloidin in NHBE treated with PBS Ctrl and EMT Supp; images taken under identical exposures). Data is representative of two donors (two inserts per donor). Scale bars: 25 µm. Polymerized actin is calculated as the summation of F-actin to total actin (G-actin+F-actin). Statistics determined by Mann–Whitney test (B,E) or Kruskal–Wallis test followed by Dunn's multiple comparison test (A), with *P*<0.05 considered statistically significant.

We have previously demonstrated that after CS exposure there is an increase in the polymerized fraction of actin in the cell, with increases in cortical tension and cell stiffness (Nishida et al., 2017). Given the cell shape changes, we reconfirmed that CS increases actin polymerization, with a similar increase in the polymerized fraction of actin in CHBE cells seen in a actin fractionation western blot assay (Fig. 5A,B) and immunofluorescence by phalloidin staining (Fig. 5C,D). As predicted, treatment of cells with Latrunculin A (LatA), which binds actin monomers with 1:1 stoichiometry and prevents them from polymerizing (Coué et al., 1987), shifts the actin ratio away from polymerized actin despite CS exposure (Fig. 5A). A dose–response curve (not shown) of LatA identified a low dose (0.25 µM) that shifts the polymerized fraction towards a ratio found in normal cells with minimal cell toxicities. In contrast, treatment with JaspA, which stabilizes or polymerizes the actin filament (Bubb et al., 1994), to increase in the fraction of polymerized actin in the CS exposed cells (Fig. 5A). No difference in polymerized actin were found in NHBE undergoing pEMT compared to PBS Ctrl (Fig. 5E,F).

### Identifying drugs to keep the epithelial cells jammed despite CS

**Fig. 6.**
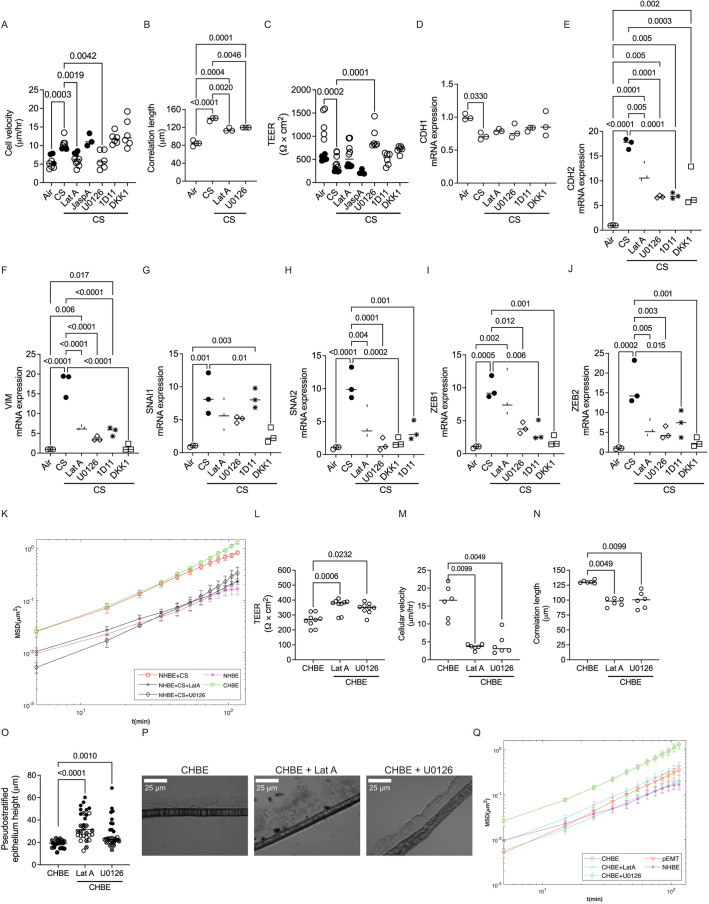
**Inhibitors of actin polymerization improve monolayer integrity and reverse plasticity.** (A–C) LatA (an actin polymerization inhibitor) and U0126 (a MAPK inhibitor) are the only drugs which reduced cellular velocity (A), TEER (B) and correlation length (C) on the CS-exposed epithelia [cells treated with pathway modulators such as LatA, the actin-polymerizing agent JaspA, U0126, the TGFβ1 neutralizer (1D11), or an antagonist of Wnt signaling pathway (DKK1) and compared to vehicle control]. Data is representative of two donors (three to six inserts per donor). (D) None of the inhibitors restored the CS-induced decrease in basal mRNA expression of the epithelial marker *CDH1*. (E–J) The levels of the mesenchymal markers (*CDH2*, *VIM*, *SNAI1*, *SNAI2*, *ZEB1* and *ZEB2*) were significantly reduced relative to GAPDH in CS-exposed epithelia treated with several pathway modulators (LatA, U0126, 1D11 and DKK1) as analyzed by qPCR. (K) LatA and U0126 treatment reduced mean square displacement (MSD) in CS exposed epithelia. Data is representative of three to six inserts from two donors. (L–O) LatA and U0126 treatment improved TEER in CHBE cells (L), LatA and U0126 treatment decreased cellular velocity in COPD epithelia (CHBE) (M), LatA and U0126 treatment reduced correlation length in CHBE cells (N), and LatA and U0126 treatment improved the height of pseudostratified epithelia on COPD epithelia (CHBE) (O). (P) Representative DIC image from three inserts from one donor taken with a 40× oil objective of CHBE treated with or without LatA or U0126 showing improved monolayer height. Scale bars: 25 µm. (Q) LatA and U0126 treatment reduced the MSD with time in CHBE cells approaching levels similar to that of NHBE. Of note, there is not a significant increase in MSD with time in pEMT-induced NHBE. Data is representative of nine inserts, except for cellular velocity and correlation length (six inserts) and height of pseudostratified epithelia (three inserts) from one donor of CHBE. Graphs are shown with median bars. Statistics determined by Kruskal–Wallis test followed by Dunn's multiple comparison test, with *P*<0.05 considered statistically significant.

We analyzed cellular jamming after treating the cells with LatA to determine whether reducing the polymerized fraction of actin would decrease cell movement and improve monolayer integrity. We also treated the CS-exposed epithelium with inhibitors of pathways that have been implicated in the development of COPD, namely inhibitors of the MAPK/ERK kinase (U0126; Favata et al., 1998), TGFβ1 (1D11; Edwards et al., 2010) and Wnt signaling pathways (DKK1; Glinka et al., 1998) . LatA and U0126 pushed the epithelia to a jammed state despite CS exposure, while the actin-polymerizing drug, JaspA, pushed cells to an unjammed state (Fig. 6A). Corresponding changes in the correlation length were observed (Fig. 6B). LatA and U0126 inhibited CS-induced barrier disruption (Fig. 6C), and did not improve mRNA *CDH1* expression (Fig. 6D), but prevented the expression of other mesenchymal and squamous-metaplasia markers (Fig. 6E–J; Fig. S2A,B). Although 1D11 and DKK1 prevented the expression of mesenchymal and squamous-metaplasia markers (Fig. 6E–J; Fig. S2A,B), neither drug inhibited the unjamming and barrier dysfunction due to CS (Fig. 6A–C). None of the inhibitors restored cilia or CBF (Fig. S2C,D). Another way to characterize the motion of cells is to compare the mean square displacements of cells, *MSD*(Δt)*.* Following (Park et al., 2015), it is defined as

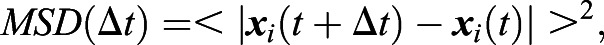
where ***x***_*i*_(*t*) is the (2D) position of cell *i* at time *t*, | | indicates absolute value, and < > indicates averaging over all times and all the cells in the culture. Hence, *MSD*(Δ*t*) represents the average displacement of cells squared over a period of time Δ*t*. The CS and CHBE cells display relatively large displacements, while the treatment of the CS-exposed cells with LatA and U0126 resulted in 3–5 times smaller displacements, similar to that of NHBE (Fig. 6K).

### Cell jamming enhances monolayer integrity in diseased epithelia

We sought to determine whether inhibition of actin polymerization (LatA) and MAPK/ERK kinase (U0126), could reverse the disruption seen in the CHBE monolayer and push the CHBE into a jammed state. We observed that LatA and U0126 repaired the epithelium (Fig. 6L), and caused a jamming effect in CHBE as quantified by cellular velocity and correlation length (Fig. 6M,N). We also saw an increase in height of the pseudostratified epithelium with the inhibition of actin polymerization and MAPK/ERK kinase (Fig. 6O,P). Similar to the results shown above, the CHBE cells display relatively large displacements, while the treatment of these cells with LatA and U0126 resulted in 3–5 times smaller displacements, to levels similar to that of NHBE (Fig. 6Q). Of note, the cells which have undergone pEMT showed MSDs only slightly greater than the NHBE (Fig. 6Q). Similar to what we found in the CS-treated cells, these inhibitors had no effect in restoring the cilia or CBF (Fig. S3A,B).

### Cofilin-1 manipulation

**Fig. 7.**
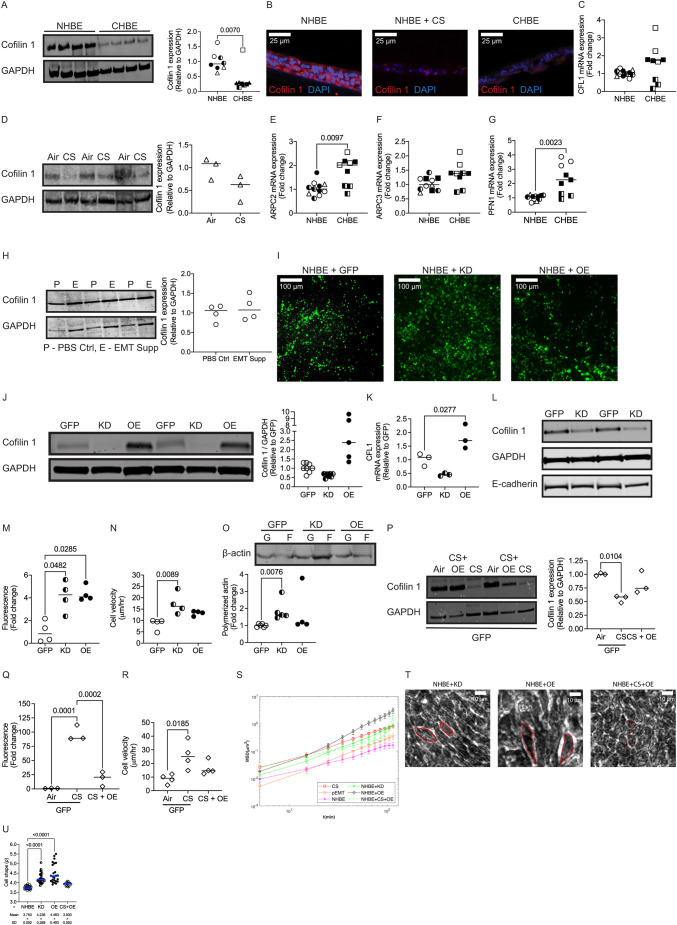
**Restoring cofilin-1 improves airway epithelial integrity.** (A) Age- and gender-matched CHBE cells have lower cofilin-1 expression (representative blot, left panel and quantification, right panel) as detected by western blotting. GAPDH levels are shown as a loading control (data is representative of four donors, one to two inserts per donor). (B) There is reduced cofilin-1 expression as seen by immunofluorescence. Representative image from four donors (two inserts per donor) of cofilin-1 (red) and DAPI (blue) in NHBE, CS-exposed NHBE and CHBE epithelia. Taken with a 40× oil objective under identical exposures. Scale bars: 25 µm. (C) Despite having lower protein expression, CHBE express higher mRNA levels of *CFL1* (encodes for cofilin-1) as analyzed by qPCR. Data is representative of three to four donors (three inserts per donor). (D) CS-exposure reduces cofilin-1 expression (representative blot, left panel and quantification, right panel) as detected by western blotting and GAPDH levels are shown as a loading control. Data is representative of three inserts from one donor. (E–G) mRNA expression of several actin-binding proteins were altered, with increases in *ARPC2*, *ARPC3* and *PFN1* as analyzed by qPCR. Data is representative of three to four donors (three inserts per donor). (H) Cells treated with EMT Supp to induce pEMT do not have decreased cofilin-1 expression (representative blots, left panel and quantification, right panel) as detected by western blotting. GAPDH levels are shown as a loading control (data is representative of four inserts from one donor). (I–U) NHBEs were transduced with adenovirus to knockdown or overexpress cofilin-1 [Ad-GFP (GFP), Ad-GFP-U6-h-CFL1-shRNA (KD) and Ad-GFP-h-CFL1 (OE) at 1×10^10^ PFU/ml]. (I) Transfection of GFP-tagged control virus, CFL1 shRNA virus, and CFL1 overexpression virus had an efficiency of ∼40–60%, as visualized by GFP fluorescence. Taken with a 20× objective. Scale bars: 100 µm. (J,K) Representative blot (left panel) and quantification (right panel) of cofilin-1 normalized to GAPDH as loading control as detected by western blotting (J), and mRNA expression of CFL1 (encodes for cofilin-1) indicating ∼50% knockdown and ∼2-fold overexpression (K). Data is representative of two to three inserts from two donors for western blotting and three inserts from one donor for qPCR. (L) Representative blot of NHBE transduced with GFP-tagged control virus (GFP), and CFL-1 shRNA virus GFP (KD) indicating that cofilin-1 manipulation did not alter E-cadherin levels. Data is representative of three inserts from one donor. (M,N) Both knockdown and overexpression of cofilin-1 increased FITC–dextran permeability (M), and knockdown increased cellular velocity, while overexpression trended towards an increase without reaching statistical significance (N). Data is generated from four inserts from one donor. (O) Cofilin-1 knockdown increased the fraction of polymerized actin (representative blot, top panel and quantification, bottom panel) of cofilin-1 expression from air and CS-exposed NHBE transduced with adenovirus as indicated, as detected by western blotting. GAPDH levels are shown as a loading control (data is representative of three inserts from one donor). (P) Cofilin-1 overexpression was sustained despite CS exposure (representative western blot, left panel and quantification, right panel) in NHBE transduced with adenovirus as indicated. Data is representative of four inserts from one donor. (Q,R) Cofilin-1 overexpression protected the monolayer from the CS-induced increase in (Q) FITC–dextran permeability, and (R) cellular velocity. Data is representative of three to four inserts from one donor. (S) Cofilin-1 over-expression protected from the CS-induced increase in MSD with time. (T) Representative images used for cell shape factor quantification in NHBE with knockdown or overexpression of cofilin-1 and in CS-exposed NHBE transduced with cofilin-1 overexpression. (U) NHBE with knockdown or overexpression of cofilin-1 demonstrated an increase in cell shape factor as compared to NHBE, whereas CS-exposed NHBE transduced with cofilin-1 overexpressing virus did not show statistical significant differences. The cell shape factor values were compared to NHBE values from Fig. 4L. Data is generated from three to four inserts from one donor. Graphs are generated with median bars. Statistics determined by Mann–Whitney test (A,C–H) or Kruskal–Wallis test followed by Dunn's multiple comparison test (J,K,M–R,U), with *P*<0.05 considered statistically significant.

We analyzed some common actin-binding proteins and found a consistent decrease in total abundance of cofilin-1 (Fig. 7A,B) despite an increase in transcript amount based on quantitative (q)PCR (Fig. 7C) in CHBE compared to that in NHBE. In addition, repetitive exposure to CS decreased cofilin-1 expression compared to air control (Fig. 7D). Although there were altered transcriptional levels of other actin-binding proteins (Fig. 7E–G), they did not result in a corresponding change in protein abundance (data not shown). Notably, we did not observe any change in total cofilin-1 expression in NHBE treated with EMT inducer supplement (Fig. 7H). Although its action is more nuanced, in general, cofilin-1 severs or breaks up actin (Bamburg and Bernstein, 2010; DesMarais et al., 2005). Therefore, we assessed whether cofilin-1 manipulation would improve monolayer integrity in CHBE and CS-exposed cells. We did so using an adenoviral transduction with evidence of corresponding changes in both protein and mRNA levels (Fig. 7I–L). We observed that E-cadherin expression was not altered due to cofilin-1 knockdown (KD) (Fig. 7L). We found an increase in monolayer permeability with both knockdown and overexpression of cofilin-1 in NHBE (Fig. 7M), without significant ciliary effects (Fig. S4A,B). KD of cofilin-1 increased the polymerized fraction of actin and increased cell velocity. While overexpression of cofilin-1 led to a trend towards increased cell velocity (Fig. 7N), this did not reach statistical significance, and there was no change in the total actin polymerization (Fig. 7O). Restoring cofilin-1 expression in CS-exposed NHBE enhanced monolayer integrity (Fig. 7P), decreased the polymerized fraction of actin (Fig. S5A) and reduced cell velocity (Fig. 7R). However, cofilin-1 did not have an effect on the percentage of pixels moving and CBF (Fig. S5B,C). Cofilin-1 KD and overexpression both increased the *MSD(*Δ*t)* (Fig. 7S). In measuring the cell shape parameter as a marker of unjamming, we found that KD of cofilin-1 elongated the cells, increasing the cell shape index, suggestive of unjamming (Fig. 7T,U). Interestingly, there was a much larger increase in the cell shape index with overexpression of cofilin-1, although these cells looked both longer and wider (Fig. 7T,U). However, with exposure to CS, cofilin-1 overexpressed cells were smaller with a cells shape index similar to that of control (Fig. 7T,U).

### CS-exposed cells and CHBE have cellular energetics that are distinct from pEMT

**Fig. 8.**
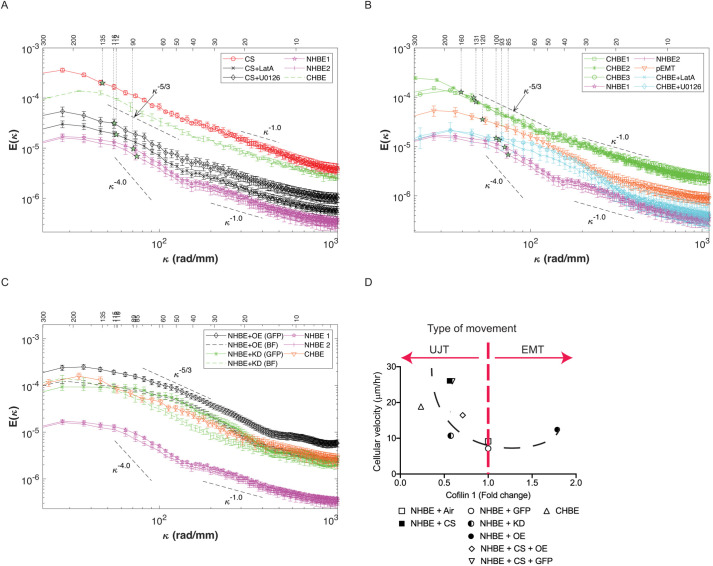
**CS-exposed epithelia and COPD epithelia have cellular energetics that are distinct from pEMT.** The COPD epithelia (CHBE) and CS-exposed NHBE were treated with LatA or U0126. The NHBE were treated with 2× EMT inducer supplement serve as a model for pEMT. (A) Ensembled average one-dimensional spatial energy spectra [E(κ), κ being wavenumber] of the horizontal velocity component is greatest for CS-exposed epithelium and CHBE, both which display classic slopes characteristic of Kolmogorov–Kraichnan turbulence as modeled by the −5/3 dashed line. Treatment of CS-exposed cells with the inhibitor of actin polymerization (LatA) or MAPK kinase inhibitor (U0126), decreased the energetics and turbulence in the monolayer approaching that of NHBE. (B) COPD epithelia (CHBE) also display classic slopes characteristic of Kolmogorov–Kraichnan turbulence, as modeled by the −5/3 dashed line. Treatment of CHBE with LatA or U0126 decreased the energetics and turbulence in the monolayer approaching that of NHBE. The pEMT cells have a completely different slope. (C) In monolayers with cofilin-1 knockdown (KD) or overexpression (OE) in NHBE, analysis was performed in the entire monolayer (brightfield image, BF) or in GFP–cofilin-1-manipulated cells only (GFP). The curve of cofilin-1 KD-specific cells (GFP) demonstrate a −5/3 slope similar to that of the CHBE cells, with similar energetics. The curve of the cofilin-1 OE-specific (GFP) cells have higher energetics, but similar slope to that seen in pEMT. (D) Biphasic relationship between cofilin-1 and cell velocity. There is a biphasic relationship of cell velocity as a function of cofilin-1 (NHBE exposed to air or cigarette smoke and treated with GFP control, KD and OE at 1×10^10^ pfu/ml and CHBE donors).

We found that the flow patterns within the various cultures consisted of multiple swirling eddies of varying sizes and magnitudes, and we sought to quantify the relationship between them and elucidate the size and energy of these eddies for the various cases. Based on known information about typical hydrodynamic motions, we calculated the one-dimensional spatial energy spectra of the velocity component in the same (*x*) direction (see Materials and Methods section) for each horizontal line of vectors, and then averaged them in time and over the *y*-direction. The error bars are calculated be performing a bootstrap analysis involving 50 subsamples of the entire database, and estimating the uncertainty as the root mean square of the deviation from the mean value. The energy spectra E(κ), shown in Fig. 8A, reflected the distribution of the horizontal contribution to the kinetic energy for varying wavenumbers (κ=2π/λ) or wavelengths (λ), namely the size of eddies. For convenience, results were presented as a function of both wavelength and wavenumber, and for clarity, the data are separated into two plots. The relative strength of eddies of different sizes could then be compared to those observed, for example, in two-dimensional (2D) turbulent flows (Kraichnan, 1971). Note that the above-mentioned correlations were, by definition, the inverse Fourier transform of these spectra; hence corresponding values are also indicated in Fig. 8A for each case. These spectra revealed several trends. First, the differences in energy among cases occurred for all eddy sizes, and the energy decreased with decreasing wavelength. Second, the weighted average wavelengths (i.e. correlation length) for the CS and three CHBE cases were higher compared to those of the other samples. In contrast, the control cases had the lowest correlation length, and their energy levels were an order of magnitude lower than those of the CHBE and CS cultures. The three CHBE spectra were very similar for most of the spectral range, except for the largest eddies [i.e. the differences in velocity magnitude and the correlation between them were only associated with the large-scale motions (>160 µm)] (Fig. 8B).

To interpret the differences between jammed to unjammed cultures, we assessed the interactions among eddies of different scales in the turbulent flow (Tennekes and Lumley, 1972; Kraichnan, 1971). These interactions are typically referred to as energy cascading, which describes the transfer of energy from eddies of a certain size to larger or smaller ones owing to merging or breakup, respectively. Unlike 3D flows, in 2D turbulent flows, energy cascading typically occurs in both directions. Upward cascading from small to larger scales owing to merging of eddies occurs in the spectral range corresponding to relatively large eddies. The energy distribution in this spectral range can be modeled by the Kolmogorov–Kraichnan scaling, E(κ)=K_ε_ε^2/3^κ^−5/3^, where K_ε_ is a constant and ε is the rate of energy transfer across scales. The distribution in this model indicates that the energy flux is scale independent and stationary; namely the rate at which energy is added to a certain size range due to merging of smaller eddies (ε) is equal to the rate at which the energy is depleted from this range due to merging into larger ones. This energy is eventually dissipated at the largest possible scales owing to interactions, e.g. with outer boundaries. In contrast, downward cascading, namely breakup of eddies to smaller ones, occurs at scales that are smaller than those at which the energy is injected. In this range, based on the model, a stationary down-cascading rate is expected to follow E(κ) of ∼κ^−3^ (Kraichnan, 1971).

As indicated by lines plotted parallel to the spectra, in the present experiments, spectra in the range of E(κ) of ∼κ^−5/3^ (with a range in slope of ±0.33) existed only for the CS (Fig. 8A) and CHBE cases (Fig. 8B) for λ>40 µm, which corresponded to cell patches with diameters larger than five cells. The interpretation from this model would suggest that the evolution of eddies larger than 40 μm in the 2D cultures behaved similarly, that is, they merged into larger ones in a scale-independent rate. Remarkably, the spectral overlap in the domain with a κ^−5/3^ slope for the three CHBE cases suggested that they started with very similar energy levels as cell patches with the characteristic size of about five cells, and had the same energy flux (merging rate) up to ∼160 µm, corresponding to ∼25 cells diameter. However, in some cells, as seen with CHBE2, the cascading process persisted to larger eddies compared to those observed for other CHBE cells. Such differences could be associated with variations in friction at the interface with the microscope or along the outer perimeter of the sample (Kraichnan, 1967), or perhaps some cell-intrinsic property that requires further dissection. However, for most of the spectral range, the distribution and energy of the eddy motions for the three CHBE cultures were very similar. The CS flow also followed a scale-independent reverse cascading process initiated at a λ of ∼40 µm. However, both the starting energy level and the subsequent kinetic energy flux were about two times higher. This difference could be associated with several factors. Some possibilities include an increase in cell membrane stiffness, which would cause the culture to be more susceptible to break up, or a decrease in the strength of adherens junctions between cells across the monolayer, which would reduce their ability to withstand stresses. In contrast, the spectra of the two NHBE cultures presented in both plots indicated that the initial energy level was about an order of magnitude smaller than that of CS or CHBE cells, and did not exhibit spectra in the range of a −5/3 slope. These trends suggest that the initial energy is too low to sustain a reverse cascading process. We also analyzed the cells that had undergone pEMT. While we did see an increase in cell velocity and correlation length in cells undergoing pEMT, the energy spectrum was distinct from what occurred in the CHBE/CS exposed cells. With pEMT, the spectrum was less energetic, with the kink occurring at a smaller scale (∼25 µm) and with a narrow reverse-cascading range with −5/3 slope, resulting in substantially lower energy at large scale. These distinctions suggest that the movement of CHBE and CS-exposed cells are distinct from the movement that occurs with mesenchymal transition.

The CHBE and CS-exposed cultures did not exhibit an E(κ) of ∼κ^−3^ range at scales smaller than that of the injected energy, which was expected for stationary breakup into small-scale eddies in a model of 2D turbulence (Kraichnan, 1971). Instead, the small-scale motions (λ<40 µm) exhibited E(κ) of ∼κ^−1^. For 2D flows, a direct transition from E(κ) of ∼κ^−5/3^ to κ^−1^ is typically observed in spectra of passive scalars (temperature or mass diffusion) where the mass diffusivity is much smaller than the viscosity (Sreenivasan, 2019; Gotoh et al., 2000). In the E(κ) of ∼κ^−1^ range, the viscosity dampened the velocity fluctuations, but the diffusivity was too small to smoothen the scalar fluctuations. Instead, the spatial variability in scalar concentration was progressively smeared by weak stresses induced by residual motions. The direct transition from E(κ) of ∼κ^−5/3^ to κ^−1^ suggested that once the initial stresses fragmented the cultures to patches with the characteristic size of five cells diameter, part of this energy (reverse) cascaded to larger scales. For the remaining energy, the residual stresses within each sub-patch were too weak to cause further fragmentation to smaller scales. Consequently, the slow energy transfer across scales causing the κ^−1^ domain could have been a result of, for example, residual stresses within each patch or stresses induced by relative motion between patches. Note that the NHBE control also exhibited a range E(κ) of ∼κ^−1^.

We compared the spectra of cofilin-1 KD and cofilin-1 overexpressing (OE) cell cultures in brightfield images that include the entire monolayer, and specific cells within the field which show protein manipulation (GFP), to that of the COPD culture. The energy levels of the GFP expressing cells and entire brightfield image of cofilin-1 KD are of the same order of magnitude as that of the COPD cells although the distributions among scales are different. The brightfield image of the cells with KD of cofilin-1 culture has a lower energy level than the GFP-expressing cells (cells which within the culture specifically have had cofilin-1 KD) at intermediate wavenumbers, with a broader region slopes in the range of −5/3, indicating better agreement with the Kolmogorov–Kraichnan turbulence. The energy of the COPD culture falls between the two measurements in the mid-wavenumber range. In contrast, while the brightfield image of the cells with cofilin-1 overexpression has an energy level comparable to that of the KD and COPD cultures, the specific GFP–cofilin-1-expressing cells are more energetic (Fig. 8C) although with slope characteristics more similar to the pEMT cells.

To better describe the effect of cofilin-1 in primary differentiated human lung epithelial cells, we assessed cell velocity in NHBE+adenoGFP, NHBE+CS+adenoGFP, NHBE+adeno-shCofilin, NHBE+adenoCofilin, NHBE+CS+adenoCofilin, and CHBE as function of cofilin-1 expression and found a biphasic relationship with the lowest velocity in control (NHBE) cells, and increased cell velocity with either increased or decreased cofilin-1 abundance (Fig. 8D). Coupling this data with the kinetic spectrum suggests an optimal cofilin-1 concentration, as seen in normal cells, allows for cellular jamming with a reduction in cofilin-1 driving the UJT with a display of collective motion, and increases in cofilin-1 allowing for mesenchymal type movement.

## DISCUSSION

Cellular plasticity, or the ability of the cell to alter its phenotype to adapt to the local environment, is critical for cellular resilience but can result in disease. Even after the removal of the injurious substance, the altered phenotype may persist, as we found with the CHBE cells.

We have quantified the phenotypic changes that occur in injured (CS-exposed) and diseased (CHBE) cells and found that although the injured/diseased cells display some features of pEMT, their cellular motion is indicative of unjamming (UJT). Transitioning to the unjammed state has been proposed as an alternative to EMT as a strategy for cellular mobility (Park et al., 2016), while others have suggested that both pEMT and UJT can co-exist (Mitchel et al., 2020). Our data suggests that with repetitive CS injury and in COPD, airway epithelial cells display a co-existence of pEMT and UJT.

We found that unjamming results from an increase in the polymerized fraction of actin, and rectifying the increase in polymerized actin pushes the monolayer back to a jammed state. There is conflicting literature on the correlation between cell stiffness and migration (Luo et al., 2016; Mihai et al., 2012; Radotić et al., 2012). In a model of asthmatic epithelium, increased stiffness reduces the ability to deform under stresses and the culture becomes more ‘brittle’ (i.e. more likely to develop cracks, as seen with glass) (Park et al., 2016), which, in the COPD epithelium is likely due to increased polymerized actin. The accompanying reduction in cell–cell contact strength, likely promotes fragmentation of the culture into migrating patches, and although untested in this study, perhaps improving adhesion strength may change the size of the migrating patches, even if it does not abrogate the movement (Vig et al., 2017). These questions will be the focus of future studies.

To alleviate these effects, we decreased the fraction of polymerized actin, using LatA, which drastically reduced the initial kinetic energy and subsequent cell migration, and improved the monolayer barrier replicating conditions in healthy epithelia. Although less robust, MAPK inhibition improves monolayer dynamics, potentially due to similar cytoskeletal mechanisms. These injured/diseased cells had a kinetic energy spectra in the range of a −5/3 slope, suggesting merging (reverse cascading) of cell patches into larger-scale eddies, and a weak/slow breakup into smaller eddies, which, based on this model, is indicative of collective migration. The higher initial kinetic energy and merging rate for the CS case compared to that of CHBE could be due to differences in the initial forcing from the external stimuli of CS. Recent studies involving cell motility, where the source of energy is at the scale of a pair of cells found the steeper spectral slopes, −4.5 in experimental data and −4 in numerical simulations (Lin et al., 2021; Angelini et al., 2010). In our study, the spectral slopes are considerably milder, about −5/3 at low wavenumbers and −1 at high wavenumbers. Such slopes are more consistent with those modeled in classical motions of passive scalars in Kolmogorov–Kraichnan turbulence, with the transition occurring at the scale of about four cells (Kraichnan, 1971; Sreenivasan, 2019; Gotoh et al., 2000). These trends suggest that motility and local interactions play less of a role in driving the motions of the present cultures, since these interactions are thought to dictate the steeper slopes (Lin et al., 2021; Angelini et al., 2010).

Comparing other pathway inhibitors implicated in COPD and with EMT (Shankar and Nabi, 2015; Xie et al., 2004; Bocci et al., 2019; Xu et al., 2009; Gasior et al., 2017), we found that manipulating the TGFβ and Wnt signaling pathway did not abrogate cell movement despite decreasing the levels of transcriptional markers of pEMT. In the CS-exposed and CHBE cells treated with LatA, the initial kinetic energy levels are too weak to generate multiscale motions analogous to turbulent flows, suggesting that the lower fraction of polymerized actin decreased the fragmentation of the monolayer. We identified a decrease in cofilin-1 as a potential mechanism to drive the increase in actin polymerization in both CS-injured cells and CHBE cells. An optimal level of cooperativity between cells is required to maintain a robust monolayer and therefore it is unsurprising that both KD and overexpression of cofilin-1 in NHBE cells increased permeability and cell velocity with evidence of a ‘Goldilocks’ level seen in normal cells, which likely reflects an actin cytoskeleton with enough mechanical plasticity to allow for stable cell–cell adhesions. The kinetic energy spectrum of the cofilin-1 overexpression cells, while higher, has a shape similar to pEMT cells with a less distinct −5/3 slope seen in CS-exposed or CHBE cells potentially, indicating that it is energetically less favorable for these cells to merge to large-scale eddies that occur with collective motion. This is particularly intriguing as others have identified increased cofilin-1 upon EMT (Haibo et al., 2017; Izdebska et al., 2018). Moreover, when we overexpressed cofilin-1 in NHBE, we see an increase in the markers of EMT. This raises the question of the role of cofilin-1 as a nodal switch in mechanisms of cellular movement. It is possible that lower levels of cofilin-1, by increasing the fraction of polymerized actin, induces UJT with monolayer fragmentation and collective motion, while higher levels may enable more individual mesenchymal type movements, although further studies are required. While both collective movements and more individual cell motion can disrupt the monolayer integrity, the strategies to rectify monolayer integrity are likely quite divergent. Future investigation of cofilin-1 localization, phosphorylation and effects on local actin dynamics, such as G-actin-to-F-actin ratios and actin branching, as well as the contribution of additional actin-binding proteins could shed further light on this biphasic relationship (Ohashi, 2015).

We propose that chronic low-grade injury causes the epithelium to undergo plasticity with co-existence of both pEMT and UJT, and that is why previously sought strategies that only address pEMT, such as manipulation of the TGF pathway, have not been successful in abrogating disease. Further investigation into mechanisms of cellular movement and levels of cofilin-1 could improve the understanding of injury/repair processes and cellular plasticity.

## MATERIALS AND METHODS

### Primary human airway epithelial cells

Primary human bronchial epithelial cells from non-diseased human bronchial epithelial cells (NHBE) and COPD-derived human bronchial epithelial cells (CHBE) were either sourced from MatTek Corporation (MA, United States) or Lonza Group AG (Basel, Switzerland) or Epithelix SàRL (Geneva, Switzerland). Demographic characteristics of the donors are shown in Table S1, with each donor being indicated with numbers 1–3 (e.g. NHBE1, CHBE1).

### Cell culture

The cryopreserved cells were expanded and maintained at an air–liquid interface (ALI) as described previously (Nishida et al., 2017; Ghosh et al., 2020). The cryopreserved cells were amplified on rat tail collagen I (Corning, NY, USA)-coated flasks with PneumaCult^TM^-Ex Plus medium (StemCell Technologies Inc., Vancouver, Canada). Cells were passaged at 80–90% confluency. At sub-confluency cells were plated onto rat tail collagen I-coated 0.4 μm pore polyethylene terephthalate clear membrane transwell inserts (Corning) with PneumaCult™-Ex Plus medium with 150,000 cells/well, 300,000 cells/well and 500,000 cells/well in 24-well, 12-well, and 6-well transwells, respectively. At 100% confluency, the transwells were put at an ALI with basolateral PneumaCult™-ALI medium (StemCell Technologies Inc., Vancouver, Canada). Cells were differentiated for 4 to 6 weeks at the ALI to obtain a fully differentiated pseudostratified epithelium.

### EMT induction in NHBE

To induce pEMT in NHBE cells at 4 to 6 weeks ALI were additionally supplemented with the basolateral 2× EMT induction supplement (StemXVivo EMT Inducing Media Supplement, R&D Systems) for 10 days.

### CS exposure

For whole CS experiments, the confluent monolayer of pseudostratified epithelium was exposed to CS in an exposure system and VC 1 manual smoking machine (Vitrocell^®^ Systems GmbH, Germany) as described previously (Nishida et al., 2017; Chandrala et al., 2019). The epithelia were exposed to one CS exposure for 10 days. One CS exposure consisted of two cigarettes (3R4F) and each burned for ∼8 min using the ISO puff regimen (one 35 ml puff every 60 s with an 8 s exhaust). The control inserts were exposed to humidified air in the exposure system.

### TEER measurement

On the apical compartment of the inserts, sterile 1× phosphate buffered saline (PBS, Thermo Fisher Scientific, NY, USA) was added. TEER was then assessed three times with an epithelial voltohmmeter (EVOM, World Precision Instruments, FL, USA) with the STX2 electrodes of 4 mm width and 1 mm thick, and a mean resistance was calculated. Values were then corrected for fluid resistance (insert with no cells) and surface area.

### Permeability

Permeability was assessed with a fluorescein isothiocyanate–dextran (FITC-dextran) flux assay as described previously (Nishida et al., 2017). Briefly 0.5 mg/ml of 4 kDa FITC–dextran (Sigma-Aldrich, MO, USA) diluted in 1× PBS was added to the apical surface of monolayer with basolateral 1×PBS. After 1 h, transwells were removed and basolateral 1× PBS were transferred to a clear bottom 96-well plate and fluorescence was measured in triplicate with an excitation of 485 nm and emission of 528 nm in a Synergy^TM^ HT multi-detection microplate reader (Biotek, VT, USA).

### CBF

Differentiated pseudostratified epithelium were placed into No. 1.5 coverslip glass top and bottom plates (MatTek Corporation, MA, USA). The plates were incubated at 37°C with 5% CO_2_ in the 3i Marianis spinning disk confocal-phase contrast microscope system (Leica Microsystems, Sugarland, TX, USA). High-speed time-lapse images were taken with a 32× air objective and a scientific CMOS camera (Hamamatsu C11440-42U30, Hamamatsu Photonics K.K., Bridgewater, MA, USA). For measuring CBF, a sequence of 250, 1800×1800 pixels images were acquired at 100 frames per second. In all the cases, the field of view was 304×304 µm^2^. A Matlab (R2018b, The MathWorks, Inc., MA, USA) program (Sisson et al., 2003) was used to determine average CBF per video. Refer to earlier studies (Kliment et al., 2021; Chandrala et al., 2019) for more details on CBF analysis.

### Cellular velocity

Cellular velocity was quantified by acquiring phase-contrast images of cell culture at a rate of one frame per 5 min for 2 h (in CBF section above) as described previously (Chandrala et al., 2019). The successive images were cross-correlated by Particle Image velocimetry using the commercial software DaVis (LaVison Inc., MI, USA) (Raffael et al., 2007). Multi-pass cross-correlation analysis with decreasing interrogation window size was used for optimizing the data quality. The size of the final correlated windows was 32×32 pixels, corresponding to 5.4 µm×5.4 µm. With a 50% overlap between the neighboring windows, the resulting spatial resolution of the vector field was 2.7 µm×2.7 µm. Vector data post-processing was performed by universal outlier detection to remove any spurious vectors (Westerweel and Scarano, 2005). For each data set, the instantaneous velocities were computed by averaging the instantaneous quantities over the entire field of view. All the instantaneous quantities were time-averaged over a period of 2 h. To quantify the intercellular coordination, the spatial autocorrelation function (Garcia et al., 2015) was determined at each time point *t* as:

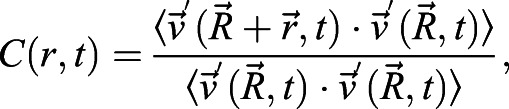
where 
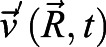
 denotes the velocity fluctuations at the position 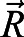, obtained by subtracting the spatial mean from the velocity field, and angular brackets indicate an average over all positions 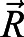. The correlation function was averaged over all directions and times. The distance where the correlation function decays to zero was the correlation length (Xi et al., 2017).

The spatial one-dimensional energy spectra of the velocity component in the *x*-direction, E(κ), was calculated from the instantaneous velocity field along a series of horizontal lines (Li et al., 2017; Hackett et al., 2009). The fast Fourier transform of the velocity field along the horizontal lines was computed after detrending and subtracting the spatially averaged velocity from each line, without any windowing functions. The instantaneous spectrum obtained for the multiple lines and times were then ensemble averaged to obtain the mean spatial energy spectra. Note that the correlation is the inverse Fourier transform of the spectrum, and represents the average length scale of the cell culture motions.

### Cell shape analysis

Time-lapse phase contrast images of cell culture at ALI were acquired as described above in cell velocity. Cells were manually traced using Fiji software (Schindelin et al., 2012) and the cell shape factor was calculated as described previously (Park et al., 2015).

### Immunofluorescence

Differentiated pseudostratified epithelium were fixed with 4% paraformaldehyde in PBS (Affymetrix Inc, OH, USA) to the apical chamber and incubated for 15 min at room temperature. The cells were washed with 1× PBS and were transferred through a series of sucrose (Sigma-Aldrich, MO, USA) infiltration steps.

The sucrose infiltration steps involved sequential incubation with sucrose solutions of 10%, 15%, 20%, 25%, and 30%, at the apical and basolateral side of inserts for 10 min. Then the membrane containing the cells was embedded in a biopsy size cryomold using Optimal Cutting Temperature (O.C.T.) Compound (Tissue-Tek, CA, USA). 10 μm sections were cut using the Leica Cryostat M 3050S (Leica Biosystem Inc., IL, USA) and attached to Superfrost Plus microscope slides (Thermo Fisher Scientific) and dried at room temperature overnight.

Slides were stained using the Shandon Sequenza Immunostaining center (Thermo Fisher Scientific). The sections were permeabilized with 0.1% Triton™ X-100 (Thermo Fisher Scientific) in 1× PBS, blocked at room temperature for 1 to 2 h with 5% bovine serum albumin (BSA, Sigma-Aldrich, MO, USA) and 10% goat serum (Thermo Fisher Scientific) in 1× PBS, and incubating with the primary antibody (see below) overnight at 4°C. Primary antibody was washed three times with 1× PBS and incubated with secondary antibody (1:200) in 1× PBS for 2 h at room temperature in the dark. After washing, the slides were mounted with ProLong® Gold antifade with DAPI (Thermo Fisher Scientific). Slides were cured overnight, sealed, and stored at 4°C for long-term storage.

Immunostaining for mouse monoclonal Na^+^/K^+^-transporting ATPase subunit alpha-1 (Na^+^/K^+^-ATPase α, 1:100, cat. no. sc-514614, Santa Cruz Biotechnology, Inc), mouse monoclonal protein kinase C (PKC ζ, 1:50, cat. no. sc-17781, Santa Cruz Biotechnology, Inc), and cofilin-1 (D3F9) XP^®^ Rabbit mAb (1:200, cat. no. 5175S, Cell Signaling Technology) was conducted. To stain for filamentous actin (F-actin), Alexa Fluor 488-phalloidin (1:200, cat. no. A12379, Thermo Fisher Scientific) staining was performed. Secondary antibody staining involved incubation with goat anti-mouse IgG (H+L), superclonal^TM^ recombinant secondary antibody, Alexa Fluor 488 (cat. no. A28175, Thermo Fisher Scientific), Alexa Fluor 555 (A28180, Thermo Fisher Scientific), Alexa Fluor 647 (A28181, Thermo Fisher Scientific), or goat anti-rabbit IgG (H+L) cross-adsorbed secondary antibody, Alexa Fluor 555 (cat. no. A21428, Thermo Fisher Scientific). The slides were imaged on a single-point, laser scanning confocal microscope (LSM 700, Carl Zeiss Microscopy LLC, NY, USA) with a 40× oil objective.

### Pseudostratified epithelium height

The height of the pseudostratified epithelium was measured manually using the line selection tool in Fiji software (Schindelin et al., 2012). A perpendicular line from the transwell membrane to the epithelium on DIC images taken with a 40× oil objective was measured. For each condition and donor, multiple regions were evaluated for each image with a range of 5 to 10 measurements.

### Transmission electron microscopy

#### Sample preparation

Samples were fixed in 2.5% glutaraldehyde and 3 mM MgCl_2_, in 0.1 M sodium cacodylate buffer (pH 7.2) overnight at 4°C. After buffer rinse, samples were fixed in 1% osmium tetroxide in 0.1 M sodium cacodylate buffer on ice in the dark for 1 h and washed with 0.1 M sodium cacodylate buffer to remove the excess fixative buffer. Samples were incubated at 4°C overnight in filtered sterile 0.1 M sodium cacodylate buffer, washed with 0.1 M maleate buffer, en bloc stained with 2% uranyl acetate (for 1 h in the dark) in 0.1 M maleate, dehydrated in a graded series of ethanol and propylene oxide, and embedded in Eponate 12 (Ted Pella) resin. Samples were polymerized at 60°C overnight. Thin sections, of 60–90 nm, were cut with a diamond knife on the Reichert-Jung Ultracut E ultramicrotome and picked up with naked 200 mesh copper grids. Grids were stained with 2% uranyl acetate (aq.) followed by lead citrate.

The samples were observed with a Philips CM120 Cryo-Electron Microscope at 80 kV and the images were captured with an AMT XR80 high-resolution (16-bit) 8 Megapixel camera (Advanced Microscopy Techniques, MA, USA).

### Scanning electron microscopy

#### Sample preparation

Samples were fixed in 2.5% glutaraldehyde and 3 mM MgCl_2_, in 0.1 M sodium cacodylate buffer (pH 7.2) overnight at 4°C. After buffer rinse, samples were fixed in 1% osmium tetroxide in buffer (1 h) on ice in the dark followed by two distilled water rinses before dehydration in ethanol. Samples were dried for SEM with hexamethyldisilazane, mounted on carbon coated stubs, and coated with 20 nm gold/palladium alloys (AuPd). The prepared samples were imaged on a Leo/Zeiss Field-emission SEM at 1 kV.

### Real-time qPCR

Total RNA was isolated from cultured primary bronchial epithelial cells and purified using the RNeasy^®^ Plus Mini Kit (Qiagen, Germany), supplemented with the Proteinase K (Qiagen, Germany) and the RNase-free DNase Set (Qiagen, Germany). cDNA of 1000 ng µl^−1^ was obtained using the High Capacity cDNA Reverse Transcription Kit (Applied Biosystems, Thermo Fisher Scientific), and the absence of DNA contamination was verified by excluding the reverse transcriptase from subsequent PCR reactions.

cDNA was subjected to PCR using the SYBR™ Green PCR Master Mix (Applied Biosystems, Thermo Fisher Scientific) to amplify epithelial (*CDH1*), mesenchymal (*CDH2*, *VIM*, *SNAI1*, *SNAI2*, *TWIST2*, *ZEB1* and *ZEB2*), and squamous metaplasia [p53 (TP53) and *IVL*] markers, mRNA encoding actin-binding proteins (ARPC2, ARPC3, CFL1 and PFN1) and *GAPDH* using primers as shown in Table S2 (Integrative DNA Technologies, IA).

Each PCR was carried out as follows: initial denaturation at 94°C for 15 min, 45 cycles of 94°C for 35 s, 60°C for 1 min and 72°C for 1 min 15 s, followed by a final extension at 72°C for 2 min. Each cycle was repeated 45 times. Based on the comparative Ct method, gene expression levels were calculated and GAPDH was used as housekeeping gene.

### Quantification of polymerized actin

The polymerization of actin was evaluated by separating the G-actin and F-actin from the differentiated epithelial cells at ALI as described previously (Nishida et al., 2017).

### Western blotting assay

Whole cells extracts were prepared with ice cold 1× RIPA buffer (Cell Signaling Technology^®^) with protease inhibitor cocktail, and phosphatase inhibitor cocktail 2 and 3 (Sigma-Aldrich). Total protein was quantified using bicinchoninic acid colorimetric assay (Pierce™ BCA Protein Assay Kit, IL, USA), separated by SDS-PAGE using the 4-15% Mini-PROTEAN^®^ TGX™ Precast Protein Gels and Mini-PROTEAN^®^ Tetra Vertical Electrophoresis Cell (Bio-Rad), transferred to PVDF (Immobilon^®^-FL PVDF membrane, Millipore Sigma) using Mini Trans-Blot^®^ Cell (Bio-Rad). Blots were incubated with primary monoclonal antibody [E-cadherin (24E10) Rabbit mAb (1:1000, Cell Signaling Technology), or cofilin-1 (D3F9) XP^®^ Rabbit mAb (1:1000, Cell Signaling Technology) or GAPDH (14C10) Rabbit mAb (1:1000, Cell Signaling Technology) or β-actin (13E5) Rabbit mAb (1:1000, Cell Signaling Technology)] and probed with secondary antibody [1:20,000, IRDye^®^ 800CW Donkey anti-Rabbit IgG (H+L) or IRDye^®^ 680CW Goat anti-Rabbit IgG (H+L) or IRDye^®^ 800CW Goat anti-Mouse IgG (H+L) or IRDye^®^ 680CW Goat anti-Mouse IgG (H+L)]. The blots were imaged by ODYSSEY CLx imaging system and quantified using Image Studio Lite (LI-COR Biosciences).

### Drug treatments

The NHBE cells at the ALI were exposed to CS for 10 days and simultaneously basolaterally treated with an antagonist of Wnt signaling pathway (recombinant human DKK1 protein, 50 ng/ml, R&D Systems), TGFβ1 neutralizer [TGFβ-1,2,3 monoclonal antibody (1D11), 1.25 μg/ml, Thermo Fisher Scientific], MAPK inhibitor (U0126, 15 μM, Cell Signaling Technology^®^), an inhibitor of actin polymerization (Latrunculin A, 0.625 μM, Sigma-Aldrich) or the actin filament-polymerizing and stabilizing agent (Jasplakinolide A, 0.25 μM, Sigma-Aldrich). Also, the CHBE at the ALI were basolaterally treated with MAPK inhibitor (U0126, 15 μM), and an inhibitor of actin polymerization (Latrunculin A, 0.625 μM) for 5 days.

### Knockdown and overexpression of cofilin-1 in NHBE

Adenovirus control (Ad-GFP), and adenovirus to knockdown cofilin-1 (Ad-GFP-U6-h-CFL1-shRNA) and express CFL1 (Ad-GFP-U6-h-CFL1) were purchased from Vector Biolabs to transduce cells. Well-differentiated NHBE at the ALI was treated apically and basolaterally with transfection medium (containing PneumaCult™-ALI medium and 5 μg ml^−1^ polybrene with Ad-GFP-U6-h-CFL1-shRNA or Ad-GFP-U6-h-CFL1 or Ad-GFP at 1×10^10^ pfu ml^−1^) for 4 h. After 4 h, the transfection medium was transferred from the apical surface to the basolateral. After 20 h, the transfecting medium was replaced with PneumaCult™-ALI medium medium. The culture was maintained for an additional 48 h.

### Overexpression of cofilin-1 in CS exposed NHBE

NHBE at the ALI were exposed to air or CS for 10 days. On 8th day of exposure, the epithelia were transfected with either Ad-GFP as a control and or with Ad-GFP-U6-h-CFL1 to overexpress cofilin-1 or Ad-GFP at 1×10^10^ pfu ml^−1^ for 48 h.

### Statistical analysis

Prism version 9.0 (GraphPad, CA, USA) was used for analysis. The results are expressed with median bars. A *P*<0.05 was considered statistically significant. Comparisons between two groups were performed using Mann–Whitney test. Kruskal–Wallis test with Dunn's multiple comparison test were performed to evaluate statistical significance for three or more groups.

## Supplementary Material

Supplementary information

Reviewer comments
