# Supplementary material for: Epithelial plasticity in COPD results in cellular unjamming due to an increase in polymerized actin
Source: J Cell Sci. 2022 Feb 24;135(4):jcs258513. doi: 10.1242/jcs.258513 (PMC8919336; doi:10.1242/jcs.258513)
Supplement: Supplementary information [file joces-135-258513-s1.pdf]

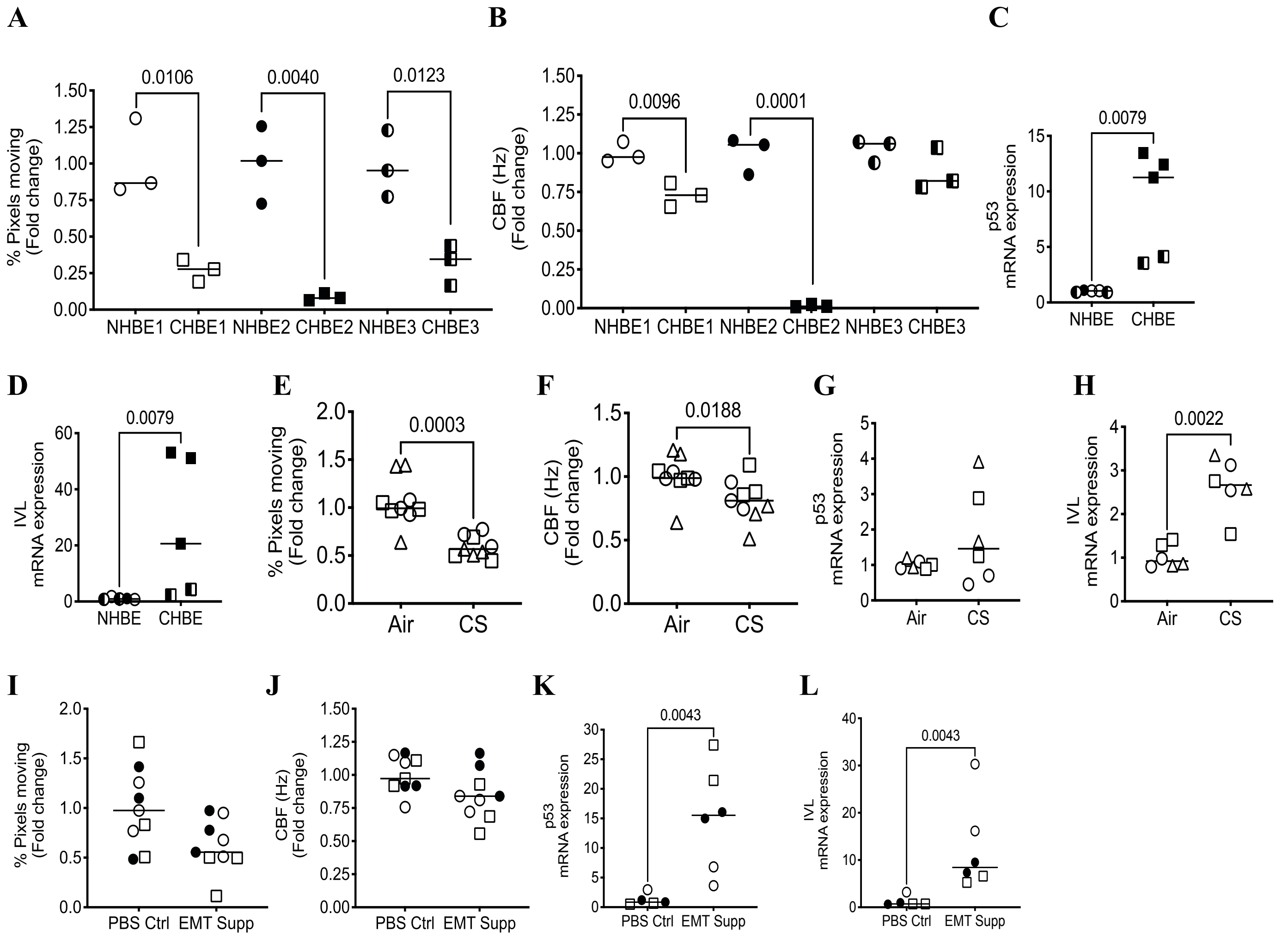

Fig. S1. COPD epithelia, cigarette-smoke exposed non-diseased epithelia, and epithelial to mesenchymal induction in non-diseased epithelia display loss of ciliary function and increased mRNA expression of squamous metaplasia related markers. (A) Percentage of moving cilia (% Pixels moving) were lower in COPD epithelia (CHBE) and **(B)** ciliary beat frequency (CBF) of CHBE were decreased as compared to gender and age-matched non-diseased epithelia (NHBE). Increased basal mRNA expression (normalized to GAPDH) of **(C)** p53 and **(D)** IVL in CHBE as compared to NHBE. **(E)** Percentage of moving cilia were lower and **(F)** CBF is decreased in cigarette-smoke (CS) exposed NHBE as compared to air exposed epithelia. Increased basal mRNA expression (normalized to GAPDH) of **(G)** p53 and **(H)** IVL in CS-exposed epithelia as compared to air control. NHBE were treated with 2X epithelial to mesenchymal transition supplement (EMT Supp.) to induce EMT and were compared to PBS Ctrl. **(I)** Percentage of moving cilia were lower in NHBE treated with EMT Supp as compared to PBS Ctrl (the difference was not statistically significant), although the **(J)** CBF was not altered in NHBE treated with EMT Supp. Increased basal mRNA expression (normalized to GAPDH) of **(K)** p53 and **(L)** IVL in primary NHBE treated with EMT Supp as compared to PBS Ctrl. Data is representative of 2 to 3 donors, 2 to 3 inserts per donor. Results are shown as median bars. Shapiro-Wilk normality test followed by Mann-Whitney test was performed. P-value < 0.05 was considered statistically significant.

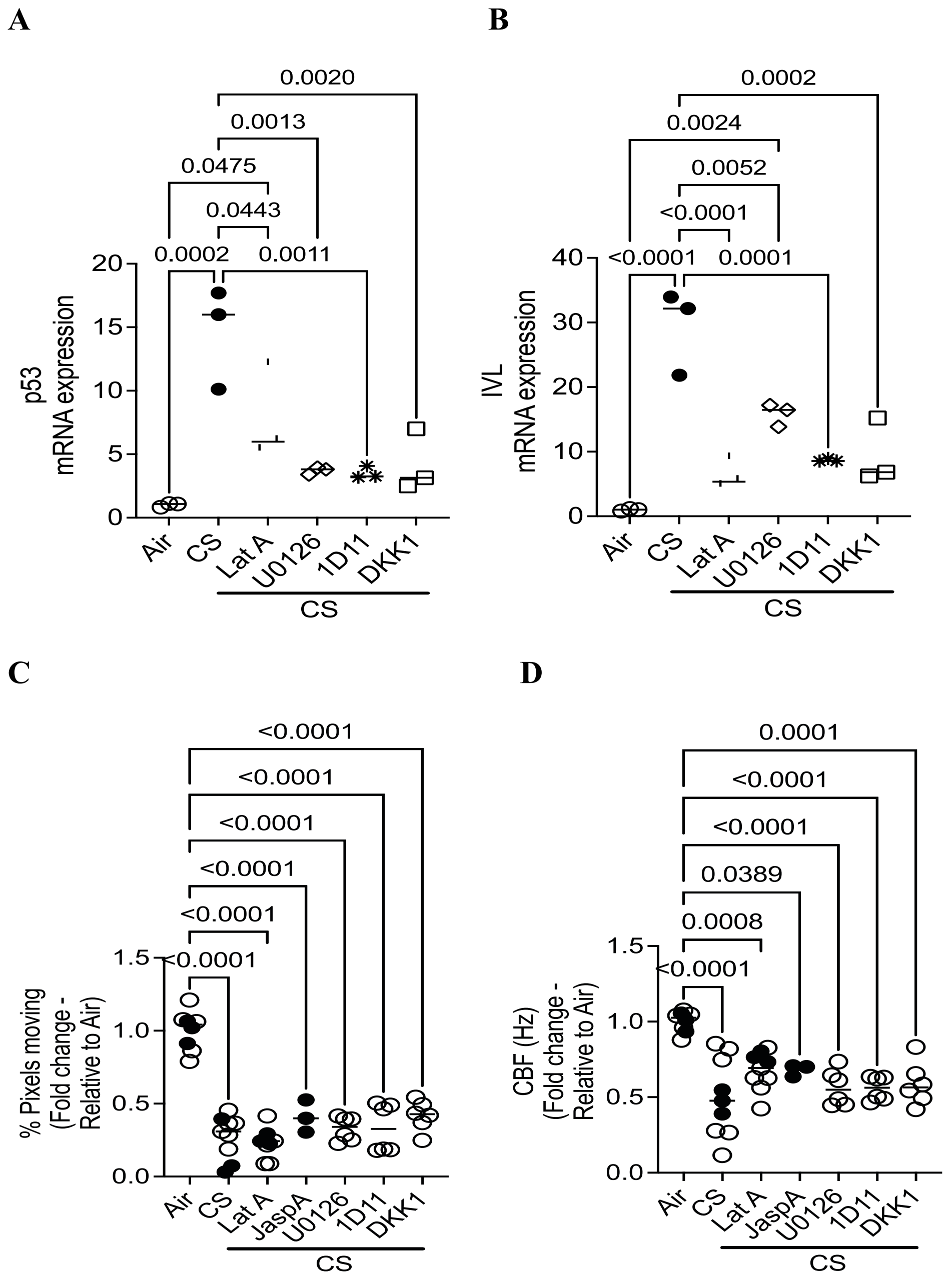

Fig. S2. All identified pathway inhibitors decreased the CS-induced expression of squamous-metaplasia markers, but do not restore CS-induced decrease in percentage of moving cilia and ciliary beat frequency. Decreased basal mRNA expression (normalized to GAPDH) of **(A)** p53 and **(B)** IVL in cigarette-smoke (CS) exposed non-diseased epithelia (NHBE) treated with actin depolymerizing agent (Lat A), MAPK kinase inhibitor (U0126), TGFβ1 neutralizer (1D11), and antagonist of Wnt signaling pathway (DKK1). However, treatment with these identified pathway inhibitors did not protect against CS induced **(C)** lower percentage of moving cilia (% Pixels moving) and **(D)** decreased ciliary beat frequency (CBF). Data is representative of 1 to 2 donor, 3 to 6 inserts per donor. Results are shown as median bars. Shapiro-Wilk normality test followed by Kruskal-Wallis test was performed. *P*-value < 0.05 was considered statistically significant.

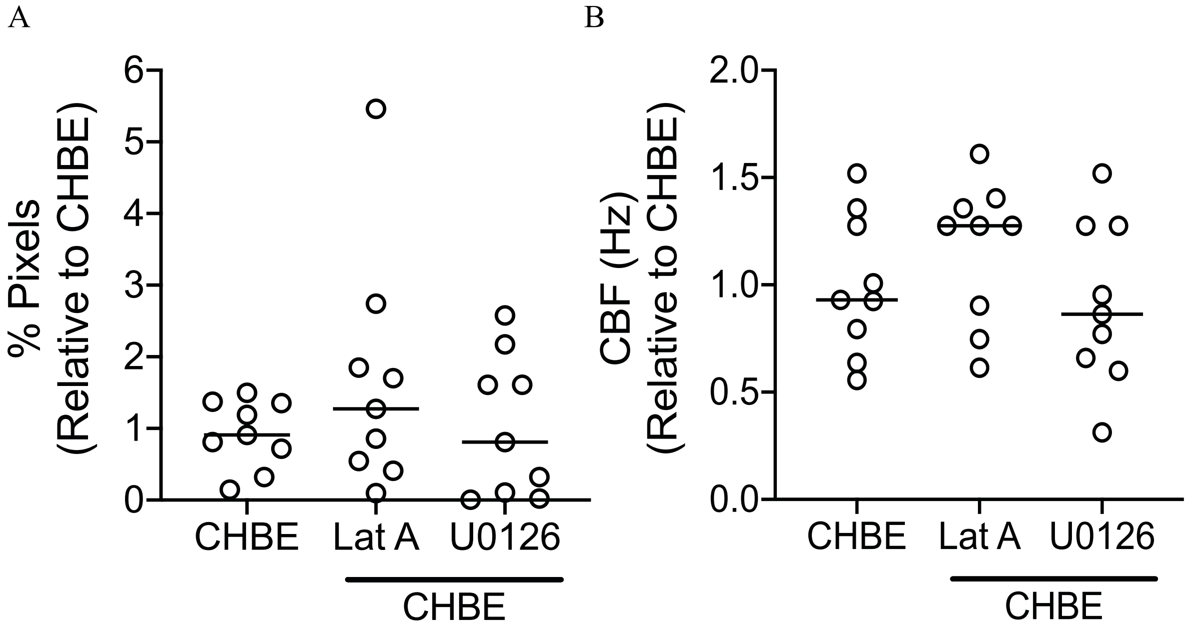

Fig. S3. Pathway inhibitors, including that of of actin polymerization and MAPK kinase pathway do not improve the percentage of moving cilia and ciliary beat frequency of COPD epithelia. Treating the COPD epithelia (CHBE) with actin polymerization inhibitor (Lat A) and MAPK kinase inhibitor (U0126) does not restore **(A)** percentage of moving cilia (% pixels) and **(B)** ciliary beat frequency (CBF). Data is representative of 9 inserts from 1 donor. Results are shown as median bars. Shapiro-Wilk normality test followed by Kruskal-Wallis test was performed. P-value < 0.05 was considered statistically significant.

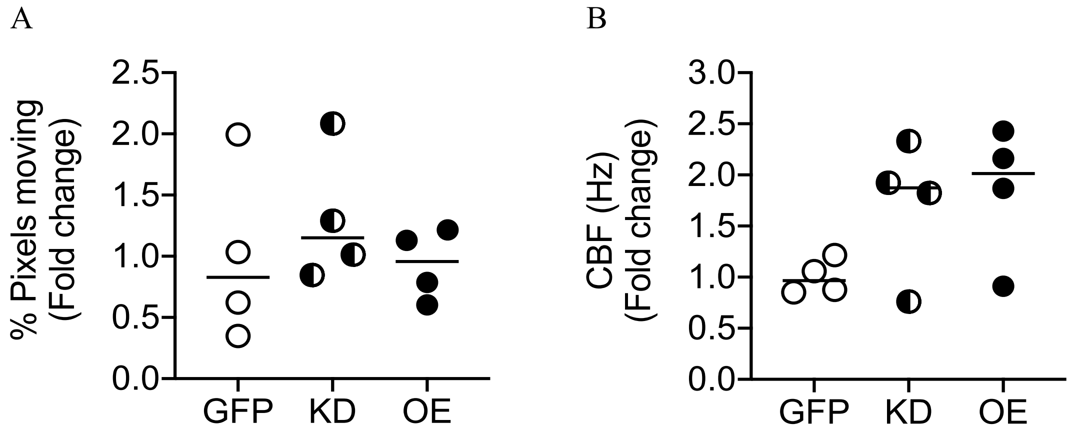

Fig. S4. Percentage of moving cilia and ciliary beat frequency is not altered with knocked down or over expressed Cofilin 1. Non-diseased epithelia (NHBE) were transduced with Adenovirus – Ad-GFP (GFP) as control or Ad-GFP-U6-h-CFL1-shRNA (KD) to knock down Cofilin 1 or Ad-GFP-h-CFL1 (OE) to overexpress Cofilin 1 at 1×10^{10} PFU/mL. The knockdown and overexpression of Cofilin 1 does not alter **(A)** Percentage of moving cilia (% pixels moving) and **(B)** ciliary beat frequency (CBF) of the NHBEs. Data is representative of 3 to 4 inserts from 1 donor. Results are shown as median bars. Shapiro-Wilk normality test followed by Kruskal-Wallis test was performed. P-value < 0.05 was considered statistically significant.

Journal of Cell Science • Supplementary information

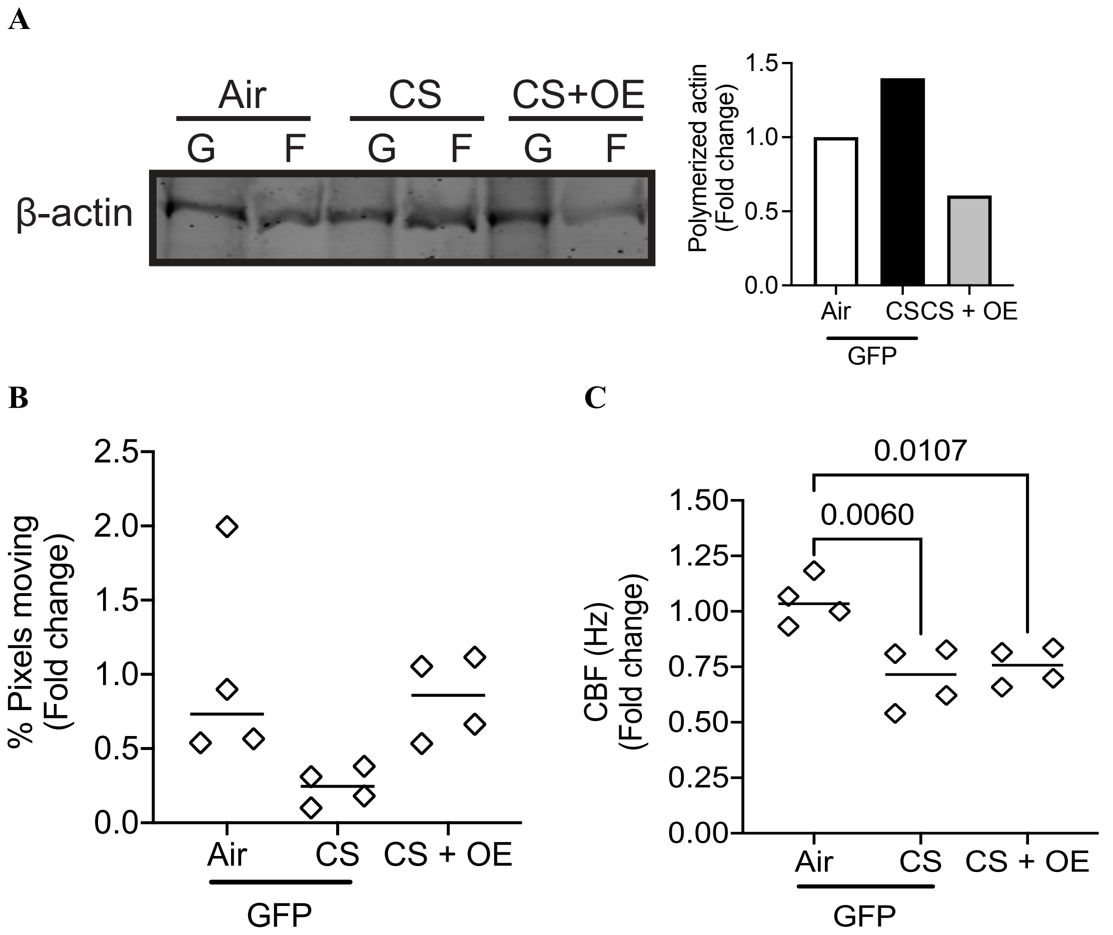

Fig. S5. Cofilin-1 overexpression preserves G-actin levels despite CS exposure but does not fully restore ciliary function. Non-diseased epithelia (NHBE) were transduced with Adenovirus – Ad-GFP (GFP) or Ad-GFP-h-CFL1 (OE) at 1×10^{10} PFU/mL prior to CS exposure. **(A)** Cofilin 1 overexpression in cigarette-smoke (CS) exposed epithelia decreased polymerized fraction due to CS exposure as shown in the representative western blot (left panel) (G: Globular actin and F: Filamentous actin) and quantification of polymerized actin (right panel). Data is representative of 2 inserts from 1 donor. **(B)** Cofilin 1 overexpression protects from the CS-induced loss of percentage of moving cilia (% pixels moving). and **(C)** Cofilin 1 overexpression in CS does not protect from the CS-induced decreased ciliary beat frequency (CBF). Data is representative of 4 inserts from 1 donor. Results are shown as median bars. Shapiro-Wilk normality test followed by Kruskal-Wallis test was performed. *P*-value < 0.05 was considered statistically significant.

Journal of Cell Science • Supplementary information

Table S1. Demographic characteristics of donors

A. Donor characteristics for age and gender matched donors

Characteristics	NHBE1	CHBE1	NHBE2	CHBE2	NHBE3	CHBE3
Age (in years)	69	67	77	79	71	64
Gender	Male	Male	Male	Male	Female	Female
Smoking history	--	Ex-smoker (25 pack-years)	--	Ex-smoker (20 pack-years)	Yes*	Yes*

B. Donor characteristics for non-diseased airway epithelia exposed to air or cigarette-smoke

Characteristics	NHBE			
	○Donor 1	▢ Donor 2	△ Donor 3	◊ Donor 4
Age (in Years)	52	62	54	65
Gender	Male	Female	Male	Female
Smoker	Yes*	No	Yes*	No
Ethnicity	Caucasian	African American	Hispanic	African American

C. Donor characteristics for non-diseased airway epithelia treated with 2X epithelial to mesenchymal inducing media supplement

Characteristics	NHBE		
	○Donor 1	▢ Donor 2	● Donor 3
Age (in Years)	52	62	54
Gender	Male	Female	Male
Smoker	Yes*	No	Yes*
Ethnicity	Caucasian	African American	Hispanic

*Pack-years data not available.
[NHBE, normal human bronchial epithelial cells; CHBE, COPD-derived human bronchial epithelial cells]

Journal of Cell Science • Supplementary information

Table S2. Primers for quantitative PCR analysis of gene transcript expression

Primer name	Sequences		Product length
	Forward	Reverse	
Housekeeping gene			
GAPDH	AACGGGAAGCTCACTGGCATG	TCCACCACCTGTTGCTGTAG	304 bp
Epithelial marker			
CDH1	CCCACCACGTACAAGGGTC	CTGGGGTATTGGGGGCATC	94 bp
Mesenchymal markers			
CDH2	CTCCATGTGCCGGATAGC	CGATTTCACCAGAAGCCTCTAC	92 bp
SNAl1	ACTATGCCGCGCTCTTTCCT	AGTCCTGTGGGGCTGATGTG	943 bp
SNAI2	TGGTTGCTTCAAGGACACAT	GTTGCAGTGAGGGCAAGAA	66 bp
TWIST2	TCTGAAACCTGAACAACCTCAG	CTGCTGTCCCTTCTCTCGAC	70 bp
VIM	GTTTCCCCTAAACCGCTAGG	AGCGAGAGTGGCAGAGGA	68 bp
ZEB1	GCTAAGAACTGCTGGGAGGAT	ATCCTGCTTCATCTGCCTGA	82 bp
ZEB2	TTTCAGGGAGAATTGCTTGA	CACATGCATACATGCCACTC	124 bp
Squamous metaplasia markers			
IVL	TGTTCCTCCTCCAGTCAATACCC	ATTCCTCATGCTGTTCCCAGTGC	227 bp
p53	GCCCAACAACACCAGCTCCT	CCTGGGCATCCTTGAGTTCC	273 bp
Transcription levels of actin binding proteins			
CFL1	GGTGCTCTTCTGCCTGAGTG	TCTTGACAAAGGTGGCGTAG	116 bp
PFN1	GTTCGTCAACATCACGCCAG	GTCCCGGATCACCGAACATT	112 bp
ARPC2	GAACCTCCTCTGGAGCTGAAAG	GAACGTGTGGATCAGGTTGATGG	132 bp
ARPC3	GAGCCTGGTTTTCCACTTCACG	GTCTCAGTCCAGTCTCTTGCCT	106 bp

Journal of Cell Science • Supplementary information

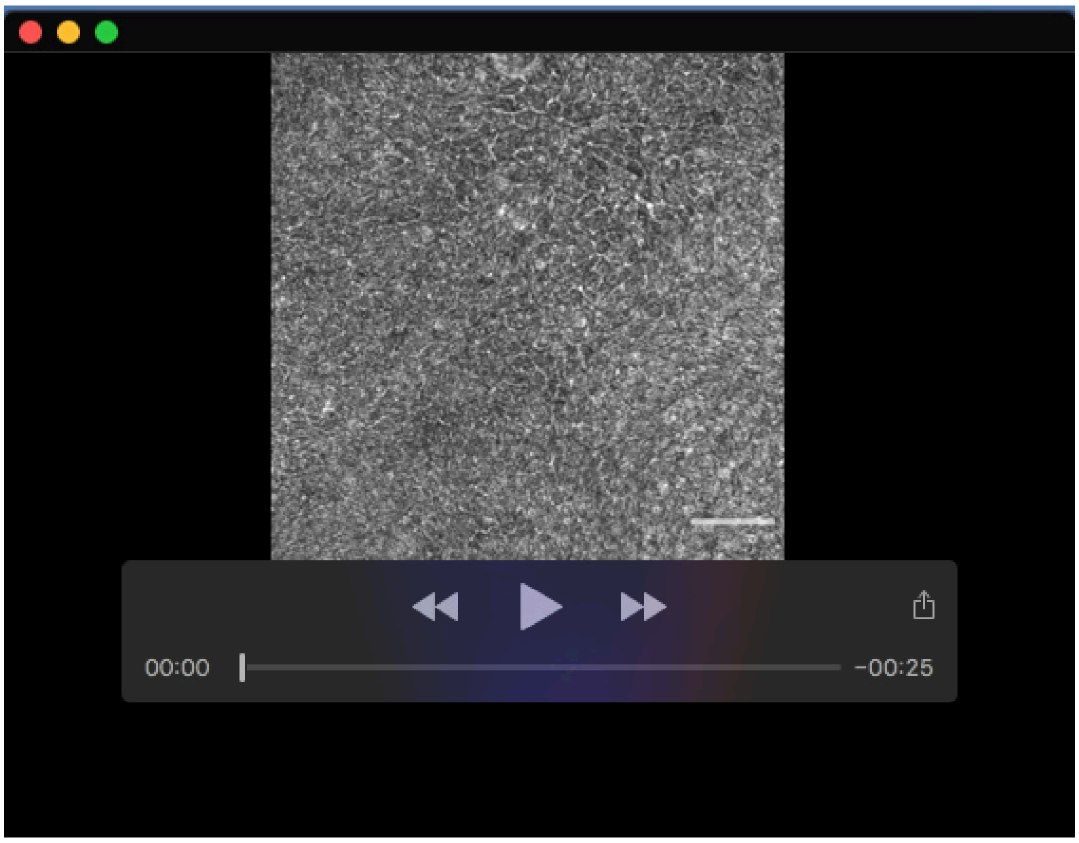

Movie 1. Representative movie of the cell migration for air exposed non-diseased epithelia (NHBE) for 10 days.

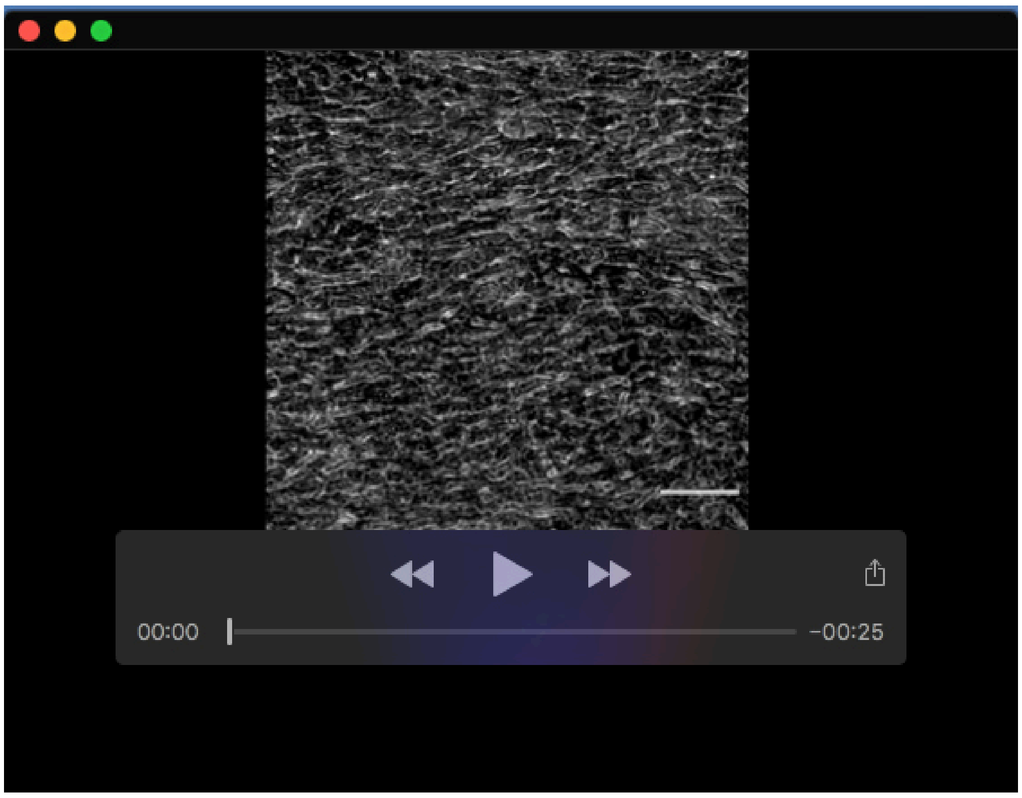

Movie 2. Representative movie of the cell migration for air exposed COPD epithelia (CHBE) for 10 days.

Journal of Cell Science • Supplementary information

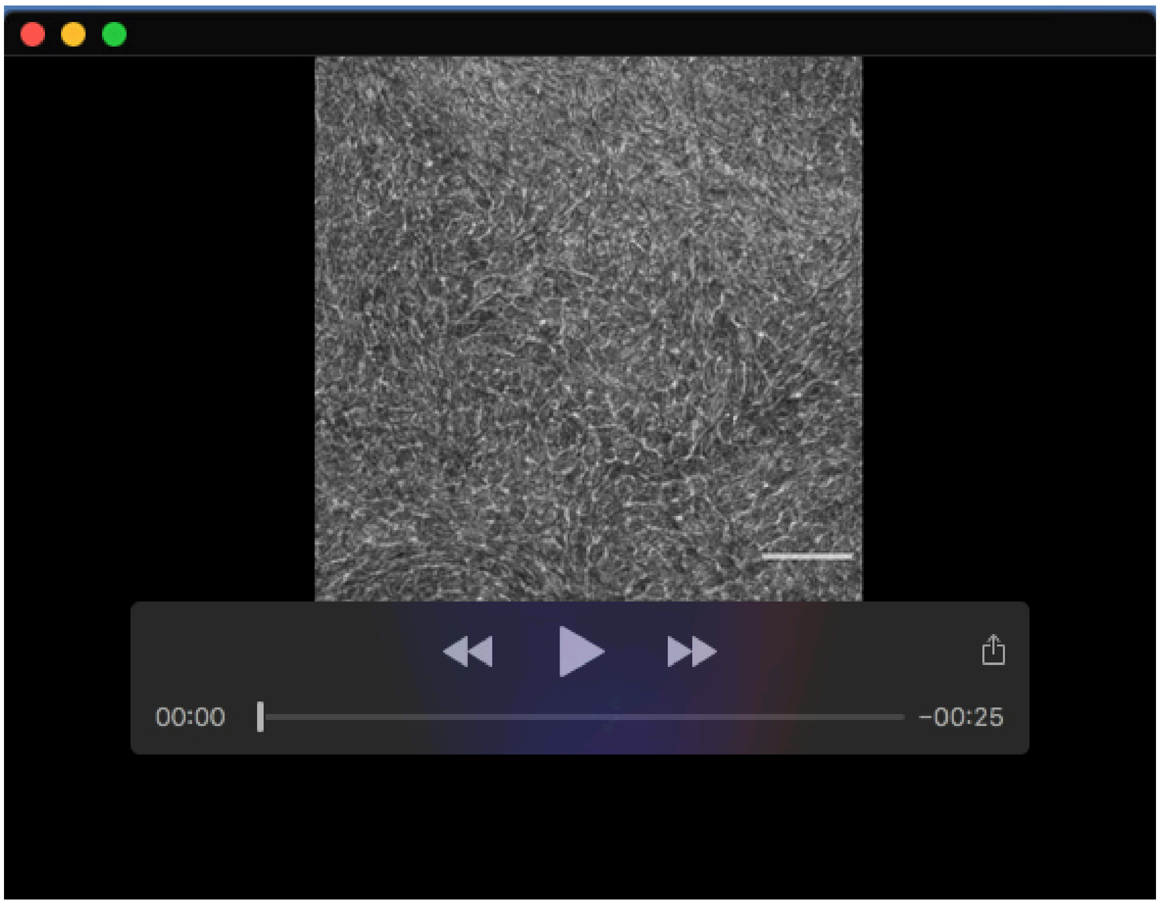

Movie 3. Representative movie of the cell migration for cigarette-smoke (CS) exposed NHBE for 10 days.

Journal of Cell Science • Supplementary information
