# Supplementary material for: Epithelial plasticity in COPD results in cellular unjamming due to an increase in polymerized actin
Source: J Cell Sci. 2022 Feb 24;135(4):jcs258513. doi: 10.1242/jcs.258513 (PMC8919336; doi:10.1242/jcs.258513)
Supplement: Reviewer comments [file joces-135-258513_review_history.pdf]

Journal of Cell Science

Epithelial plasticity in COPD results in cellular unjamming due to an increase in polymerized actin

Baishakhi Ghosh, Kristine Nishida, Lakshmana Chandrala, Saborny Mahmud, Shreeti Thapa, Carter Swaby, Si Chen, Atulya A. Khosla, Joseph Katz and Venkataramana Sidhaye
DOI: 10.1242/jcs.258513

Editor: Kathleen Green

Review timeline

Original submission:	8 February 2021
Editorial decision:	23 March 2021
First revision received:	6 October 2021
Editorial decision:	10 November 2021
Second revision received:	24 November 2021
Editorial decision:	22 December 2021
Third revision received:	27 December 2021
Accepted:	4 January 2022

Original submission

First decision letter

MS ID#: JOCES/2021/258513

MS TITLE: Epithelial plasticity in COPD results in cellular unjamming due to cell-stiffness

AUTHORS: Baishakhi Ghosh, Kristine Nishida, Lakshmana Chandrala, Saborny Mahmud, Shreeti Thapa, Carter Swaby, Si Chen, Atulya A Khosla, Joseph Katz, and Venkataramana Sidhaye
ARTICLE TYPE: Research Article

We have now reached a decision on the above manuscript.

To see the reviewers' reports and a copy of this decision letter, please go to: https://submit-jcs.biologists.org and click on the 'Manuscripts with Decisions' queue in the Author Area. (Corresponding author only has access to reviews.)

As you will see, the reviewers have divergent opinions, but overall raise a number of substantial criticisms that prevent me from accepting the paper at this stage.

In particular I would direct your attention to comments of referee #2, who states that a similar concept is well appreciated in airway cells for asthmatics; thus, the conclusions made here in the context of COPD are less novel from a general cell biology perspective. They go on to say that what is potentially most interesting to cell biologists is that toggling a particular pathway might reverse the motile state of these cells and induce/restore the jammed state. But again here novelty and thoroughness of analysis are questioned. While pulmonary biologists might find the cofilin-rescue experiment sufficiently compelling, the role for cofilin in cell motility across a range of cell systems is well-appreciated for cell biologists. Another point made by more than one reviewer is that changes in 'stiffness'¤ in your system are only inferred here. Lastly, the authors missed an opportunity to cross-correlate spatial features in the epithelium with functional read-out/cooperativity. For all these reasons the paper would need substantive additional experiments

© 2022. Published by The Company of Biologists under the terms of the Creative Commons Attribution License (https://creativecommons.org/licenses/by/4.0/).

and revision to be reconsidered for JCS. You may want to consider submitting to a different journal to minimize time to publication, and if so, let us know.

If you think that you can deal satisfactorily with the criticisms on revision, I would be amenable to see a revised manuscript. We would then return it to the reviewers.

We are aware that you may be experiencing disruption to the normal running of your lab that makes experimental revisions challenging. If it would be helpful, we encourage you to contact us to discuss your revision in greater detail. Please send us a point-by-point response indicating where you are able to address concerns raised (either experimentally or by changes to the text) and where you will not be able to do so within the normal timeframe of a revision. We will then provide further guidance. Please also note that we are happy to extend revision timeframes as necessary.

Please ensure that you clearly highlight all changes made in the revised manuscript. Please avoid using 'Tracked changes' in Word files as these are lost in PDF conversion.

I should be grateful if you would also provide a point-by-point response detailing how you have dealt with the points raised by the reviewers in the 'Response to Reviewers' box. Please attend to all of the reviewers' comments. If you do not agree with any of their criticisms or suggestions please explain clearly why this is so.

Reviewer 1

Advance summary and potential significance to field

On several scores, this report is challenging and is complex. First, it is really two papers rolled into one. One paper deals with the biology of injury and its relationship to unjamming of the airway epithelium. The other deals with novel ideas concerning the physics of energy transfer in a turbulent unjammed epithelial layer. Second, the role of cofilin in epithelial plasticity is seen as being central but paradoxical, as described below. Finally, Mitchel et al (2020) showed that the UJT and the EMT are conceptually distinct, but the predicted that in certain circumstances, such as development of injury, the UJT and pEMT may coexist and work independently, sequentially, or interactively (1). The cases here of CS and COPD seem to substantiate those predictions.

The paper would benefit, and become far more readable, if these distinctions were made clear at the outset.

Comments for the author

On several scores, this report is challenging and is complex. First, it is really two papers rolled into one. One paper deals with the biology of injury and its relationship to unjamming of the airway epithelium. The other deals with novel ideas concerning the physics of energy transfer in a turbulent unjammed epithelial layer. Second, the role of cofilin in epithelial plasticity is seen as being central but paradoxical, as described below. Finally, Mitchel et al (2020) showed that the UJT and the EMT are conceptually distinct, but the predicted that in certain circumstances, such as development of injury, the UJT and pEMT may coexist and work independently, sequentially, or interactively (1). The cases here of CS and COPD seem to substantiate those predictions.
The paper would benefit, and become far more readable, if these distinctions were made clear at the outset.
THE PARADOXICAL ROLE OF COFILIN: Cofilin is widely regarded to be an actin severing protein; in the papaper this is not made clear. All other factors being equal, less cofilin therefore implies more polymerized actin and therefore greater cell stiffness. The injured epithelium is shown here to express less cofilin, more polymerized actin, and, inferentially, stiffer cells (although cell stiffness was not measured). The authors argue that restoring cofilin-1 in the CS-exposed cells pushes the epithelium back towards a jammed state and improves monolayer integrity; the implication is that softer cells promote jamming, whereas stiffness cells promote unjamming and disruption of monolayer integrity. They go on to show that unjamming per se has been associated

© 2022. Published by The Company of Biologists under the terms of the Creative Commons Attribution License (https://creativecommons.org/licenses/by/4.0/).

with no change on monolayer integrity, so something else must be going on in addition, such as the partial EMT
Cellular plasticity is pivotal in the resilience of the epithelia. More cofilin, as occurs in the uninjured, jammed epithelium implies greater mechanical plasticity of the cell itself. However, mechanical plasticity of the cellular collective is ordinarily associated with the unjammed phase. In this case, therefore, the role of cofilin in plasticity at the cellular versus the multicellular level seems paradoxical. It would be helpful if the authors were to make that paradox more clear.
COEXISTENCE OF EMT AND UJT: It might help understanding and flow of the manuscript if you would frame it in the context of the predictions of Mitchel et al, as noted above.
TURBULENT ENERGY TRANSFER: This could easily be a stand-alone paper. Here however, it stands out like a sore thumb. Better framing in the abstract and introduction may help the flow of the paper.
In this connection, on page 11 the authors state that energy is eventually dissipated at the largest possible scales. This statement conflicts with my understanding of the turbulent energy cascade, wherein energy cascades predominantly from the largest to the smallest spatial scales. It also conflicts with the well-known poem about turbulence by Lewis Fry Richardson:

“Big whirls have little whirls,
That feed on their velocity;
And little whirls have lesser whirls,
And so on to viscosity.”

About energy cascades in unjammed 2D epithelial systems, the authors should take into account the new report on this same topic(2): Lin et al. https://dx.doi.org/10.1038/s42005-021-00530-6
Page 6 bottom: Disrupted epithelial barrier function and adherens junction proteins in CHBE and cigarette smoke (CS) exposed non-diseased epithelia
This is confusing. Is not the CBHE by definition a disease epithelium?
Page 8. “Given the changes in cell shape and expression of EMT markers, we sought to determine if these altered cellular mobility.”
“these”has an ambiguous antecedent.
Page 13: Unjamming of cells is associated with increase in polymerized actin.
This is consistent with the finding of Kim et al that unjammed epithelial systems exhibit greater traction forces. (3-5)

General Figure legends are all cryptic. For each panel, please add in the legend the central finding that the reader is to appreciate.

1. J. A. Mitchel et al., In primary airway epithelial cells, the unjamming transition is distinct from the epithelial-to-mesenchymal transition. Nature communications 11, 5053 (2020).
2. S.-Z. Lin, W.-Y. Zhang, D. Bi, B. Li, X.-Q. Feng, Energetics of mesoscale cell turbulence in two-dimensional monolayers. Communications Physics 4, (2021).
3. J. H. K. Jin-Ah Park1*, Dapeng Bi2, Jennifer A. Mitchel1, Nader Taheri Qazvini1,8, Kelan Tantisira3, Chan Young Park1, Maureen McGill1, Sae-Hoon Kim1, Bomi Gweon1, Jacob Notbohm1, Robert Steward, Jr.1, Stephanie Burger1, Scott H. Randell4, Alvin T. Kho5, Dhananjay T. Tambe1,9, Corey Hardin1, Stephanie A. Shore1, Elliot Israel3, David A. Weitz6, Daniel J. Tschumperlin7, Elizabeth P. Henske3, Scott T. Weiss3, M. Lisa Manning2 James P. Butler1, 3, Jeffrey M. Drazen1, Jeffrey J. Fredberg1, Unjamming and cell shape in the asthmatic airway epithelium. Nature Materials, (2015).
4. J. H. Kim et al., Unjamming and collective migration in MCF10A breast cancer cell lines. Biochemical and Biophysical Research Communications 521, 706-715 (2020).
5. J. H. Kim et al., Propulsion and navigation within the advancing monolayer sheet. Nat Mater 12, 856-863 (2013).

Jeffrey Fredberg

Reviewer 2

Advance summary and potential significance to field

Epithelial plasticity in COPD results in cellular unjamming due to cell stiffness

Rationale: This study aims to understand how airway epithelial cells respond to inhaled toxicants (e.g., cigarette smoke).

© 2022. Published by The Company of Biologists under the terms of the Creative Commons Attribution License (https://creativecommons.org/licenses/by/4.0/).

In previous work (Nishida et al, 2017), this group showed that smoke could alter barrier function of airway epithelial cells by reducing levels of E-cadherin/b-catenin proteins, as well as shifting actin towards a polymerized state, leading to tension induced reductions in barrier function. Remarkably, similar cellular phenotypes are seen in cells isolated from patients with COPD, a smoking-related disease, suggesting that smoking can lead to persistent changes in gene/protein expression that impact epithelial properties. Absent clear therapeutic targets for COPD, the current study seeks to deepen understanding of how airway epithelia respond to cigarette smoke. Findings: Authors find that cigarette smoke phenocopies media conditions that induce a partial EMT (e.g., decreased TEER/increased barrier leak, decreased ciliary movement, pseudo-stratification, changes in classic EMT markers at mRNA level) (Figs1-3). Cigarette smoke also inhibits cell jamming, leading to a more motile state, which the authors extensively quantify using PIV-type analyses to characterize cooperativity of cell movements (Fig. 4). Authors again reinforce similarity between epithelia from COPD and short-term cigarette exposure. Based on the authors' previous work that cigarette smoke shifts actin into the filamentous state, this study uses a low dose of latrunculin to normalize/"rescue" a more jammed epithelial state (based on motility correlation length quantification approach from Fig. 4), despite presence of cigarette smoke and even in cells from COPD patients (Fig. 5-6). Lastly, the authors show that cofilin 1 protein levels are substantially reduced by cigarette smoke or in airway cells from COPD patients, where transfection/add-back reduces cell velocities/increases jamming—although the authors acknowledge a biphasic contribution of cofilin, where highest overexpression levels associate with greater cell velocities (Fig. 7). The authors appear to rule out roles for other pathways previously implicated in cell motility, such as TGFb/Wnt signaling, although evidence that pathway inhibitors are working as intended is not shown.

Assessment: This study clearly shows that cigarette smoke treated airway epithelial cells and cells derived from COPD patients manifest a similar un-jammed phenotype- where the methods used to quantify this phenotype appear robust.
While possibly novel in the COPD field, this concept is well appreciated in airway cells for asthmatics. What is potentially most interesting to cell biologists- is the notion that toggling a particular pathway might reverse the motile state of these cells and induce/restore the jammed state. It is here where I am not sure that the story is either sufficiently novel or robustly analyzed. For example, I think that pulmonary biologists might find the cofilin-rescue experiment sufficiently compelling, but for general cell biologists, the role for cofilin in cell motility across a range of cell systems is long established. I also think the authors have to be careful invoking changes in "stiffness" in their system, when it is only inferred from bulk changes in F-actin and not actually measured. Lastly, while I like the use of PIV-based data-rich analyses—it seems the authors missed an opportunity to cross-correlate spatial features in the epithelium with functional read-out/cooperativity—rather that rely on qPCR/immunoblot analyses that pool/average data across an entire filter of cells.

Specific:
1. Control: Would be nice to see the GFP-expression in the cofilin KD and overexpression experiments particularly in the monolayer cultures (how many cells infected, cofilin staining along-side), given the modestness of the immunoblot in Fig. 7F.

Comments for the author

see above

<u>Reviewer 3</u>

Advance summary and potential significance to field

This manuscript describes an unjamming transition seen in cultured human bronchial epithelial layers due to exposure to cigarette smoke, and also in cultures from patients with COPD. The authors identify qualitative features of an unjamming transition in the presence of those perturbations that do not occur in control systems or in a model system for the epithelial to mesenchymal transition (EMT). To quantify some of these qualitative results, the authors characterize the kinetic energy spectra of the tissue and compare it to that in turbulent flow. To attempt to identify some of the biological mechanisms that drive this unjamming transition, the

© 2022. Published by The Company of Biologists under the terms of the Creative Commons Attribution License (https://creativecommons.org/licenses/by/4.0/).

authors show an increase in polymerized actin, which they hypothesize to increase cell stiffness and drive unjamming. They also show a decrease in Cofilin-1 (actin-binding protein) that may account for the increase in actin polymerization. Importantly, they demonstrate that artificially increasing Cofilin-1 can rescue the jamming transition associated with healthy tissue.

Overall, this is an interesting manuscript that fits within the scope of the Journal of Cell Science. It is really interesting that the authors can quantitatively demonstrate a change in epithelial barrier function, which is correlated with a quantitative change in the collective behavior of the epithelial sheet, when cells are exposed to cigarette smoke or come from a COPD patient. The fact that they can connect this to at least one specific molecular mechanism (actin polymerization) and rescue the collective phenotype with Cofilin-1 is especially exciting. For these reasons, the manuscript achieves the bar for broad interest and impact required for publication in JOCES.

Comments for the author

There are a few major concerns/questions that should be addressed before the manuscript is published, and we also highlight some small-scale suggestions/concerns.

Major points:

1) A major statement made in the manuscript is that features of the kinetic energy spectra are an important metric describing the fluidity of the tissue, which can be compared across samples. Specifically, it is claimed that the distribution of eddy sizes is important, just as in turbulent flow. Unfortunately, the rationale for this comparison is not made clear, and evidence for a cascade of energy flow across scales is not there. In particular, the evidence for power-law scaling in experimental data is incredibly weak. It seems unnecessarily complicated, too. From my perspective, it is not at all clear that the energy spectra add anything beyond the correlation length analysis, and should be dropped entirely. Here are some sub-questions to emphasize these points:

a. What are the error bars on the estimation of the power law for the energy scaling in Fig 4? It appears that the -5/3 scaling occurs over less than half a decade of data on both the x- and y-axes, which is far below the bar of what is normally required to indicate a true scaling relation.

b. What is the error bar on the data itself for the low wavelength data in panels G in Fig 4) below about 50 microns? In other words, I'm assuming the kinetic energy is extracted from displacement fields between microscope images, and so noise in the images and error in the image cross-correlation method will give rise to a noise in the displacement fields and corresponding noise in the energy spectra. How large is this noise, as it should be more important at the smaller wavelengths?

c. Isn't an equally valid interpretation of the energy spectra data that there is a characteristic length scale for velocity correlations (which may change in CS and COPD systems compared to "normal" tissues), and then there is just noise at smaller scales? There are good reasons to expect that large-scale vortices with a characteristic lengthscale will emerge in tissues, see for example [Henkes, Silke, et al. "Dense active matter model of motion patterns in confluent cell monolayers." Nature communications 11.1 (2020): 1-9.] There is no prior evidence for turbulent flow in epithelia (though there is in other systems like bacteria where velocities are a lot higher and constituent objects are a lot smaller and have interactions with surrounding fluid), it is difficult to understand how turbulence would arise given the length and timescales involved, and the data here is not convincing.

d. I don't think the paper needs to interpret this data using a turbulence-based perspective in order to be interesting and worth publishing.

2) Although the authors emphasize an increase in cellular stiffness drives the unjamming transition, it is well known that actin and myosin behavior is tightly coupled to the behavior of adhesive molecules important in epithelia, especially E-Cadherin, via for example Rho/Rac pathways, e.g. [Yamada, Soichiro, and W. James Nelson. "Localized zones of Rho and Rac activities drive initiation and expansion of epithelial cell–cell adhesion." Journal of Cell Biology 178.3 (2007): 517-527.]. Therefore, it would be useful to understand if the actin polymerization is truly the driver of the unjamming transition, or whether upstream or downstream changes to adhesion are driving the transition instead. Is it possible to quantify or knockdown Ecad under the various conditions studied in this paper?

3) The authors mention that unjamming in the absence of changes in cell density implicate a change in cell shape. This is important, as previous work cited here by Park et al emphasizes that

© 2022. Published by The Company of Biologists under the terms of the Creative Commons Attribution License (https://creativecommons.org/licenses/by/4.0/).

cell shape changes are associated with the unjamming transition in this culture system. But here there are no direct measurements of cell shape. While it is obviously going to be difficult to use a watershed algorithm or similar to extract cell shape in an automated fashion from these images, it should be possible to examine cell shapes by hand in a representative sample. Does the cell shape change across the unjamming transition? Can that be associated with a change to actin polymerization? This is really important to explain precisely how the implicated molecular mechanism (actin polymerization) drives the unjamming transition. Since it has already been shown that cell shape changes can drive an unjamming transition, if the authors can show that altering actin polymerization alters cell shape, that would be a very high impact, important advance. Right now, there is a big gap.

Minor comments:
1. Typos
a) Page 3, first paragraph, first line: "repetitive" is spelled wrong
b) Page 4, second paragraph, second line: "expression of a cell-cell adhesion proteins" - remove "a".
c) Page 7, last line: " although not all of the changes are in line with mesenchymal transition." -"the mesenchymal transition"
d) Page 8, last paragraph, second line: "either a delay in the formation of jammed-like state" - "a jammed-like state"
e) Page 9, first paragraph, second last line: "These data indicated that the insult could cause the cells transition to a collective movement demonstrating in a fluid-like behavior" - "cause the cells to transition to a collective movement demonstrating a fluid-like behavior"
f) Page 9, second paragraph, first sentence. I think you are referring to Fig4D not Fig4G.
g) Page 12, first paragraph: "We also analyzed the cells which have undergone pEMT, to determine if their cellular motion and they energy spectrum" - "their energy spectrum"
h) Page 13, last paragraph: - "performed" is spelled wrong
i) Page 16, first paragraph: "resembles" is spelled wrong
j) Page 19, first paragraph: "However, his raises the question" - "However, this raises the question"

2. Please define COPD in the abstract and the first time it's used in the paper.
3. A connection to the work by Park et al in which HBECs undergo a rigidity transition from shape changes during maturation could be explored using your controls. They observed that asthmatic cells became spontaneously jammed at a significantly later time than the controls. Does increasing actin polymerization of HBECs affect the timing of this spontaneous jamming transition? Does the addition of CS delay the transition?
4. Adding other measures of fluidity such as mean-squared displacement or rearrangement rate would also help connect it the recent literature in the field.
5. The quality of some of the figures is low making them blurry and hard to read. Maybe try and save them as vector images. Including: Figure 2,3,4,5,6 6. Please label each subplot of each figure with its own letter.
7. The P-values shown in figure 5J are difficult to parse.
8. It's not clear how the % pixel moving plots are measured or used.
9. It isn't always clear what the authors mean by cellular plasticity and the definition in the first line of the paper could be improved on.

First revision

Author response to reviewers' comments

Dear Green,

Thank you for taking our manuscript into consideration for publication in *Journal of Cell Science*, and the invitation to submit a revised manuscript for review. We want to thank the Reviewers for their time, thoughtful comments, and overall support for our manuscript. We appreciate that the manuscript is complex and from these reviews, recognize of the information and novelty of our

© 2022. Published by The Company of Biologists under the terms of the Creative Commons Attribution License (https://creativecommons.org/licenses/by/4.0/).

findings may have been lost in our presentation. As such, we have significantly reorganized the manuscript and hope that this new flow will highlight the novelty of our findings and improve its readability. We have addressed each comment below, with our responses in blue. With the incorporated suggestions and changes, we feel that manuscript is greatly improved in its clarity.

Reviewer 1 Comments for the Author:
On several scores, this report is challenging and is complex. First, it is really two papers rolled into one. One paper deals with the biology of injury and its relationship to unjamming of the airway epithelium. The other deals with novel ideas concerning the physics of energy transfer in a turbulent unjammed epithelial layer. Second, the role of cofilin in epithelial plasticity is seen as being central but paradoxical, as described below. Finally, Mitchel et al (2020) showed that the UJT and the EMT are conceptually distinct, but the predicted that in certain circumstances, such as development of injury, the UJT and pEMT may coexist and work independently, sequentially, or interactively (1) . The cases here of CS and COPD seem to substantiate those predictions. The paper would benefit, and become far more readable, if these distinctions were made clear at the outset.

We thank the reviewer for his thoughtful read of the manuscript and appreciate that as written, it was challenging to review. We have significantly reorganized the manuscript to better integrate the two ideas that were raised in this review. The biology of injury and disease does indicate an increase in cellular motility due to an increase in the polymerized fraction of actin, which results from reduced cofilin-1 levels. The physics of energy transfer suggests two important points:
1) that they energy spectrum of the UJT is distinct from that of pEMT, allowing for additional quantitative evidence of how these biologic processes are distinct although it is highly possible that one could transition to another, and 2) that both low and high cofilin-1 levels play a role in mediating cell motility, albeit through distinct processes leading to a differential energy cascade.

Although outside the scope of this already dense manuscript, this is likely because of localized cofilin-1 concentration on the actin cytoskeleton.

1. THE PARADOXICAL ROLE OF COFILIN: Cofilin is widely regarded to be an actin severing protein; in the paper this is not made clear. Cofilin is widely regarded to be an actin severing protein; in the paper this is not made clear. All other factors being equal, less cofilin therefore implies more polymerized actin and therefore greater cell stiffness. The injured epithelium is shown here to express less cofilin, more polymerized actin, and, inferentially, stiffer cells (although cell stiffness was not measured). The authors argue that restoring cofilin-1 in the CS-exposed cells pushes the epithelium back towards a jammed state and improves monolayer integrity; the implication is that softer cells promote jamming, whereas stiffness cells promote unjamming and disruption of monolayer integrity.

All other factors being equal, less cofilin therefore implies more polymerized actin and therefore greater cell stiffness. The injured epithelium is shown here to express less cofilin, more polymerized actin, and, inferentially, stiffer cells (although cell stiffness was not measured). The authors argue that restoring cofilin-1 in the CS-exposed cells pushes the epithelium back towards a jammed state and improves monolayer integrity; the implication is that softer cells promote jamming, whereas stiffness cells promote unjamming and disruption of monolayer integrity. They go on to show that unjamming per se has been associated with no change on monolayer integrity, so something else must be going on in addition, such as the partial EMT. Cellular plasticity is pivotal in the resilience of the epithelia. More cofilin, as occurs in the uninjured, jammed epithelium implies greater mechanical plasticity of the cell itself. However, mechanical plasticity of the cellular collective is ordinarily associated with the unjammed phase. In this case, therefore, the role of cofilin in plasticity at the cellular versus the multicellular level seems paradoxical. It would be helpful if the authors were to make that paradox more clear.

We appreciate the reviewer for pointing the subtlety of the mechanical landscape and appreciate the input to improve the clarity of the manuscript. Furthermore, the reviewer is correct in that we are inferring that the cells are stiffer in this manuscript, however this inference is based on our published measurements of cell stiffness using both micropipette aspiration (MPA) and magnetic twisting cytometry (MTC) showing that repetitive exposure to CS increases epithelial cell stiffness (Nishida et al. 2017). But to be more precise in this manuscript, instead of referring to an increase in

© 2022. Published by The Company of Biologists under the terms of the Creative Commons Attribution License (https://creativecommons.org/licenses/by/4.0/).

cell stiffness, we have edited the text to refer to an increase in polymerized actin. It is possible the paradox of the role of cofilin-1 reflects the balance of conditions of cellular cooperativity required to maintain cellular jamming with optimal cell-cell adhesions. We have tried to emphasize this more in the discussion.

2. COEXISTENCE OF EMT AND UJT: It might help understanding and flow of the manuscript if you would frame it in the context of the predictions of Mitchel et al, as noted above.
We appreciate the co-existence of these features and have edited the manuscript to highlight this. Interestingly, it appears that actin polymerization primarily affects UJT, as specialized apical features such as ciliary function were not restored.

3. TURBULENT ENERGY TRANSFER: This could easily be a stand-alone paper.
Here, however, it stands out like a sore thumb. Better framing in the abstract and introduction may help the flow of the paper.

We apologize for the confusion and have edited the manuscript in response to the suggestions of this review. We do think the reason for incorporating the kinetic energy spectra into this manuscript is that is substantiates the fundamental difference in the movement seen with low cofilin-1 levels and the UJT and high cofilin-1 levels and pEMT. We believe that the kinetic energy indicates a measurable characteristic of cells in pEMT that is more a functional readout than transcriptional analysis.

4. In this connection, on page 11 the authors state that energy is eventually dissipated at the largest possible scales. This statement conflicts with my understanding of the turbulent energy cascade, wherein energy cascades predominantly from the largest to the smallest spatial scales. It also conflicts with the well-known poem about turbulence by Lewis Fry Richardson: “Big whirls have little whirls,
That feed on their velocity;
And little whirls have lesser whirls, And so on to viscosity.”
About energy cascades in unjammed 2D epithelial systems, the authors should take into account the new report on this same topic (2): Lin et al (2021).

We appreciate the reminder about Richardson’s (1922) famous description of the energy cascading process in turbulent flows. One of the co-authors has cited the same poem in a review article about sub grid-scale modeling more than 20 years ago (Meneveau and Katz, 2000 *Annual Review of Fluid Mechanics*, vol. 32: 1-32). However, the comment overlooks the fact that there is a fundamental difference between two-dimensional (2D) and three dimensional (3D) turbulent flows. While forward cascading, i.e., fragmentation of eddies from large scales to smaller ones, is the dominant phenomenon in three-dimensional (3D) turbulence, this is not the case in two- dimensional (2D) flows. As proven and discussed at length in numerous publications, some of them provided in the paper (references 2D turbulence), starting from the seminal theoretical work by Kraichnan in 2D turbulent flows, energy is injected at a certain scale and then cascades in both directions, i.e., forward to smaller scales, and backward (referred to inverse or reverse cascading) to larger scales. This means that in 2D flows, it is common for eddies to merge to form larger ones and so on. This turbulence is typically referred to as Kolmogorov-Kraichnan Turbulence and is mentioned in the paper by Lin et al. recommended by the reviewer.

As such, we would also like to thank the reviewer for the Lin et al. (2021) reference about mesoscale turbulence in 2D monolayers. This paper was published after our original manuscript was submitted so we were not aware of it. We refer to this paper in the revised manuscript. Interestingly, this paper discusses the difference between their measured energy spectra which have a slope of $k^{-4.5}$ (measured) and k^{-4} (modeled), and those of the Kolmogorov-Kraichnan turbulence. The latter has a slope of –1 at high wave numbers, and –5/3 at low wave numbers, as mentioned in the Lin et al. paper as well. As discussed in the classical paper about 2D turbulence (Tennekes and Lumley, 1972; Kraichnan, 1971), the energy is injected at the transition between these domains, and the reverse cascading occurs in the wavenumber range where the slope is -5/3. Lin et al. conclude that in their case, the steeper slope is one of the indications that cell-cell local/social interactions (local alignment and contact inhibition of locomotion) and cell motility play dominant roles in their cultures, hence they do not behave like Kolmogorov- Kraichnan

© 2022. Published by The Company of Biologists under the terms of the Creative Commons Attribution License (https://creativecommons.org/licenses/by/4.0/).

turbulence. In contrast, in our case, the spectra (Figure 8) clearly have wavenumber ranges with slopes of -5/3 and -1, i.e., consistent with the Kolmogorov-Kraichnan turbulence. These findings suggest that self-propelled motions due to active cell-cell interactions do not play a primary role. These observations are highlighted in the revised manuscript. We have edited the discussion to reflect this (Page 19, lines 404 – 406).

5. Page 6 bottom: Disrupted epithelial barrier function and adherens junction proteins in CHBE and cigarette smoke (CS) exposed non-diseased epithelia. This is confusing. Is not the CBHE by definition a disease epithelium?

We apologize for the confusion. Yes, CHBE is a disease epithelium. We were showing that both injury of normal epithelium with CS and disease epithelium in this section but will clarify in the text.

6. Page 8. “Given the changes in cell shape and expression of EMT markers, we sought to determine if these altered cellular mobility.” “these” has an ambiguous antecedent.
We apologize for the ambiguity and have edited the text accordingly to reflect that we sought to determine if the cell shape altered motility.

7. Page 13: Unjamming of cells is associated with increase in polymerized actin. This is consistent with the finding of Kim et al that unjammed epithelial systems exhibit greater traction forces. (3-5)
We thank you for directing our attention to this and have cited this reference in the text.

General
Figure legends are all cryptic. For each panel, please add in the legend the central finding that the reader is to appreciate.

We apologize for the confusion and have edited the legends accordingly.

Reviewer 2 Advance Summary and Potential Significance to Field:

Reviewer 2 Comments for the Author:

Overall Assessment: This study clearly shows that cigarette smoke treated airway epithelial cells and cells derived from COPD patients manifest a similar un-jammed phenotype- where the methods used to quantify this phenotype appear robust. While possibly novel in the COPD field, this concept is well appreciated in airway cells for asthmatics. What is potentially most interesting to cell biologists- is the notion that toggling a particular pathway might reverse the motile state of these cells and induce/restore the jammed state. It is here where I am not sure that the story is either sufficiently novel or robustly analyzed. For example, I think that pulmonary biologists might find the cofilin-rescue experiment sufficiently compelling, but for general cell biologists, the role for cofilin in cell motility across a range of cell systems is long established.

We appreciate that there are several similarities to studies previously published, but there are some key findings that are unique. The COPD cells have delay/failure in jamming that as the reviewer points out is reversed by cofilin-1 rescue. However, we would insist that there are several novel aspects of the manuscript that we believe are of interest to cell biologists. First, as highlighted by another reviewer, quantitatively demonstrate a change in epithelial barrier function in response to external injury or with disease, which is correlated with a quantitative change in the collective behavior of the epithelial sheet, is a fundamental aspect of cell biology. Moreover, that this is occurs due to altered actin polymerization and the collective phenotype is rescued by cofilin-1 is noteworthy. Moreover, we believe that from a cell biological perspective is in the biphasic effect of cofilin-1, which as another reviewer pointed out appears to be paradoxical from a mechanical plasticity standpoint. It is not just that rescue of cofilin-1 decreases motility but that both lower and higher amounts of cofilin-1 lead to mobility, but the type of mobility is different based on the cofilin-1 level as demonstrated by the kinetic energy. In analyzing the energy with the motion, we can distinguish cell behaviors that are dependent on proteins that are not just evident

© 2022. Published by The Company of Biologists under the terms of the Creative Commons Attribution License (https://creativecommons.org/licenses/by/4.0/).

based on cell velocity alone and we believe that this does provide a new understanding of cellular function as a consequence of cofilin-1. We hope that we have better communicated these aspects in our revised manuscript.

1) The authors appear to rule out roles for other pathways previously implicated in cell motility, such as TGFb/Wnt signaling, although evidence that pathway inhibitors are working as intended is not shown.
In Fig. 6E-J, we show qPCR to show that EMT markers went down with manipulation of the TGFb/Wnt signaling pathway implicating pathway inhibition, but without an effect on cell movement. We have made this point clearer in both the figure legends and text.

2) I also think the authors have to be careful invoking changes in “stiffness” in their system, when it is only inferred from bulk changes in F-actin and not actually measured.

We do agree that this is inferred in this manuscript and therefore have changed our description of increased polymerized actin instead of cell stiffness in this work to be more precise. However, we did invoke this change in stiffness based on our previous published findings using the same system, where we showed that repetitive CS exposures both increase the polymerized fraction of actin and increase cortical tension and stiffness as measured by both MPA and MTC (Nishida et al. 2017).

3) Lastly, while I like the use of PIV-based data-rich analyses—it seems the authors missed an opportunity to cross-correlate spatial features in the epithelium with functional read-out/cooperativity—rather that rely on qPCR/immunoblot analyses that pool/average data across an entire filter of cells.

As recommended by the reviewers, we have expanded the motion analysis, and the revised version contains an expanded comparison of energy spectra and mean square displacement for the different cases. Figure 8C (compares the spectra of cofilin-1 knock down (KD) and over expressed (OE) cell cultures, both looking at the entire monolayer (BF) and the subsection of cells that have evidence of protein manipulation (GFP), to that of the COPD culture. As is evident, the energy level of the cells KD culture is of the same order as that of the COPD although the distribution among scales is slightly different. The COPD culture has less energy in the midsize range, and more energy at large scales. Such small differences could be caused e.g. by variations in time evolution or comparisons between data obtained at different times. The entire KD culture has a lower energy level at intermediate wavenumbers, with a broader region with -5/3 slope, indicating a better agreement with the Kolmogorov-Kraichnan turbulence.

4) Specific:
Control: Would be nice to see the GFP-expression in the cofilin KD and overexpression experiments, particularly in the monolayer cultures (how many cells infected, cofilin staining along-side), given the modestness of the immunoblot in Fig. 7F.

While there is some variability, we do have an efficiency of about 40-60% based on immunofluorescence (Fig. 7I), which is probably contributing to the modestness in the monolayer change. Confluent primary cells are particularly challenging to transduce, and hence require viral vectors, but even so, certain proteins are difficult to manipulate.

Reviewer 3 Advance Summary and Potential Significance to Field:

This manuscript describes an unjamming transition seen in cultured human bronchial epithelial layers due to exposure to cigarette smoke, and also in cultures from patients with COPD. The authors identify qualitative features of an unjamming transition in the presence of those perturbations that do not occur in control systems or in a model system for the epithelial to mesenchymal transition (EMT). To quantify some of these qualitative results, the authors characterize the kinetic energy spectra of the tissue and compare it to that in turbulent flow. To attempt to identify some of the biological mechanisms that drive this unjamming transition, the authors show an increase in polymerized actin, which they hypothesize to increase cell stiffness and drive unjamming. They also show a decrease in Cofilin-1 (actin-binding protein) that may account for the increase in actin polymerization. Importantly, they demonstrate that artificially increasing Cofilin-1 can rescue the jamming transition associated with healthy tissue.

© 2022. Published by The Company of Biologists under the terms of the Creative Commons Attribution License (https://creativecommons.org/licenses/by/4.0/).

Overall, this is an interesting manuscript that fits within the scope of the Journal of Cell Science. It is really interesting that the authors can quantitatively demonstrate a change in epithelial barrier function, which is correlated with a quantitative change in the collective behavior of the epithelial sheet, when cells are exposed to cigarette smoke or come from a COPD patient. The fact that they can connect this to at least one specific molecular mechanism (actin polymerization) and rescue the collective phenotype with Cofilin-1 is especially exciting. For these reasons, the manuscript achieves the bar for broad interest and impact required for publication in JOCES.
We appreciate the reviewer's interest in our manuscript and are glad that they feel that it achieves the bar required for publication in *Journal of Cell Science*.

Reviewer 3 Comments for the Author:
There are a few major concerns/questions that should be addressed before the manuscript is published, and we also highlight some small-scale suggestions/concerns.

Major points:

1) A major statement made in the manuscript is that features of the kinetic energy spectra are an important metric describing the fluidity of the tissue, which can be compared across samples. Specifically, it is claimed that the distribution of eddy sizes is important, just as in turbulent flow. Unfortunately, the rationale for this comparison is not made clear, and evidence for a cascade of energy flow across scales is not there. In particular, the evidence for power-law scaling in experimental data is incredibly weak. It seems unnecessarily complicated, too. From my perspective, it is not at all clear that the energy spectra add anything beyond the correlation length analysis and should be dropped entirely. Here are some sub-questions to emphasize these points:

We apologize for the confusion in our presentation. We have reorganized the manuscript to better communicate our ideas. Our data suggests that the kinetic energy spectra distinguish movement seen with pEMT from that which occurs with cellular unjamming with CS or in CHBE. Moreover, the amount of cofilin-1 in the cell affects this movement. As a result, we do believe there is benefit in including it in this analysis and hope our reorganization of the data will better communicate these ideas.

2) What are the error bars on the estimation of the power law for the energy scaling in Fig 4? It appears that the -5/3 scaling occurs over less than half a decade of data on both the x- and y-axes, which is far below the bar of what is normally required to indicate a true scaling relation.

With the restructuring we have reorganized the figures, and the energy scaling is now in Fig. 8. We have added error bars to all the energy spectra. They are calculated by performing a bootstrap analysis involving fifty subsets of the entire database. The uncertainty is estimated as the root mean square of the deviation from the mean value. It is true that the -5/3 slope range is rather narrow, inherent to the range of scales possible in the culture. However, given how different the present trends are from e.g., those presented recently for motile cells by Lin et al. (2021), where the slope is in the -4.0 to -.4.5 range, the present agreement with the classical Kolmogorov-Kraichnan spectrum is striking. The present trends suggest that in our cultures cell-cell interactions and motility do not play a major role in the culture dynamics.

3). What is the error bar on the data itself for the low wavelength data in panels G in Fig 4) below about 50 microns? In other words, I'm assuming the kinetic energy is extracted from displacement fields between microscope images, and so noise in the images and error in the image cross-correlation method will give rise to a noise in the displacement fields and corresponding noise in the energy spectra. How large is this noise, as it should be more important at the smaller wavelengths?

As described in the response to the previous question, we have added error bars to all the spectra. Owing to the many factors that may affect the error in measurements, including the correlation-based analysis of particle displacement, for each case, we estimate the uncertainty based on a bootstrap analysis involving 50 subsets of the entire database. Consistent with the reviewer's comment, the relative uncertainty increases with wavenumber (decreasing wavelength). Yet, the

© 2022. Published by The Company of Biologists under the terms of the Creative Commons Attribution License (https://creativecommons.org/licenses/by/4.0/).

observed trends and their agreement with the classical Kolmogorov-Kraichnan spectrum persist. Hence, we respectfully disagree and believe that the signal to noise ratio of the present analysis is sufficient for supporting the claimed trends.

4). Isn't an equally valid interpretation of the energy spectra data that there is a characteristic length scale for velocity correlations (which may change in CS and COPD systems compared to "normal" tissues), and then there is just noise at smaller scales? There are good reasons to expect that large-scale vortices with a characteristic lengthscale will emerge in tissues, see for example [Henkes, Silke, et al. "Dense active matter model of motion patterns in confluent cell monolayers." Nature communications 11.1 (2020): 1-9.] There is no prior evidence for turbulent flow in epithelia (though there is in other systems like bacteria where velocities are a lot higher and constituent objects are a lot smaller and have interactions with surrounding fluid), it is difficult to understand how turbulence would arise given the length and timescales involved, and the data here is not convincing.

Several recent papers refer to the flow in cell cultures as "turbulence", e.g. Lin et al. (2021) and Angelini et al. (2010), which have been added as new references. Furthermore, in Lin et al. (2021) in is argued that because of cell-motility and cell-cell interactions, their spectra deviate significantly from the Kolmogorov-Kraichnan model. Their measured and modeled spectral slopes are -4.5, and -4, respectively. The present results are more consistent with those of the classical model for passive 2D turbulence, suggesting that cell-cell interactions are not the dominant chanisms driving the flow. To highlight the significance of the presently observed trends, we have added the following discussion to the revised paper.

Interestingly, a recent study involving cell-cell local interactions and cell motility, hence the source of energy is at the scales of a pair of cells, the spectral slopes are steeper, -4.5 in experimental data and -4 in numerical simulations (Lin et al. 2021). This is clearly not the case in the present cultures, where the spectral slopes are -5/3 at low wavenumbers and -1 at high wavenumbers, consistent with the classical Kolmogorov-Kraichnan turbulence, with the transition occurring at the scale of about four cells. Hence, motility and local interactions do not seem to play a primary role in the present cultures.

5) I don't think the paper needs to interpret this data using a turbulence-based perspective in order to be interesting and worth publishing.

We are very appreciative that the reviewer finds our paper to be worthwhile even in the absence of all the included data. As discussed in the response to the previous question, the motion of cell cultures has been analyzed using a turbulence-based perspective in several recent papers. We believe that the analogy made in our paper to the classical 2D turbulence, in contrast to the spectra observed for motile cultures, is insightful.

6) Although the authors emphasize an increase in cellular stiffness drives the unjamming transition, it is well known that actin and myosin behavior is tightly coupled to the behavior of adhesive molecules important in epithelia, especially E-Cadherin, via for example Rho/Rac pathways, e.g. [Yamada, Soichiro, and W. James Nelson. "Localized zones of Rho and Rac activities drive initiation and expansion of epithelial cell-cell adhesion." Journal of Cell Biology 178.3 (2007): 517-527.]. Therefore, it would be useful to understand if the actin polymerization is truly the driver of the unjamming transition, or whether upstream or downstream changes to adhesion are driving the transition instead. Is it possible to quantify or knockdown Ecad under the various conditions studied in this paper?

We agree with this point and have already shown the E-cadherin is decreased with CS and in COPD (Nishida et al. 2017). Cell-cell adhesion also plays a role and decreasing this will allow the cells to move. We believe there is a balance between cell stiffness and cell adhesion and altering either will disrupt this balance and work to increase UJT. Decreasing cell stiffness and increasing cell adhesion are both strategies to push the cells into a jammed state. We have recently submitted a separate manuscript outlining the effects of E-cadherin in the lung. However, as suggested we have examined the effects of cofilin knockdown on E-cadherin and see that it is unchanged (Fig. 7J).

7) The authors mention that unjamming in the absence of changes in cell density implicate a

© 2022. Published by The Company of Biologists under the terms of the Creative Commons Attribution License (https://creativecommons.org/licenses/by/4.0/).

change in cell shape. This is important, as previous work cited here by Park et al emphasizes that cell shape changes are associated with the unjamming transition in this culture system. But here there are no direct measurements of cell shape. While it is obviously going to be difficult to use a watershed algorithm or similar to extract cell shape in an automated fashion from these images, it should be possible to examine cell shapes by hand in a representative sample. Does the cell shape change across the unjamming transition? Can that be associated with a change to actin polymerization? This is really important to explain precisely how the implicated molecular mechanism (actin polymerization) drives the unjamming transition. Since it has already been shown that cell shape changes can drive an unjamming transition, if the authors can show that altering actin polymerization alters cell shape, that would be a very high impact, important advance. Right now, there is a big gap

As suggested by the reviewer, we have examined the cell shapes by hand in several representative samples and do see a change in shape that is reflective of findings that Park et al. have observed with a cell shape parameter in the range that would indicate cell unjamming (Fig. 4K).
We do believe that this change in cell shape is due to an increase in actin polymerization with a change in cell stiffness, as we have previously shown. Our data indicates this is due to a decrease in cofilin-1. And the cumulative change in cell shape likely effects monolayer height, as we have measured.

6) Minor comments:

1. Typos
We thank the reviewer for identifying these, and we have addressed them.

2. Please define COPD in the abstract and the first time it's used in the paper. We thank the reviewer for identifying these, and we have addressed this.

3. A connection to the work by Park et al in which HBECs undergo a rigidity transition from shape changes during maturation could be explored using your controls. They observed that asthmatic cells became spontaneously jammed at a significantly later time than the controls. Does increasing actin polymerization of HBECs affect the timing of this spontaneous jamming transition? Does the addition of CS delay the transition?

We appreciate the point raised by the reviewer and have included data with Jasplakinolide (JaspA) in the manuscript (Fig. 6A,C). Since these cells are primary differentiated epithelium, as opposed to the undifferentiated cells studied by Park et al, they start at a jammed state before treatment with the drug, so we cannot comment on delay in jamming. However, increasing actin polymerization does cause the cells to reverse the transition back to an unjammed state. CS treatment does the same.

4. Adding other measures of fluidity such as mean-squared displacement or rearrangement rate would also help connect it the recent literature in the field.

We appreciate the reviewer's suggestions and have added mean-squared displacement data in Fig 6K, 6Q, and 7Q).

5. The quality of some of the figures is low making them blurry and hard to read. Maybe try and save them as vector images. Including: Figure 2,3,4,5,6 .

Thank you for the suggestion. We were having difficulty in reducing the size of the files for submission, and this was helpful.

6. Please label each subplot of each figure with its own letter.

We have edited accordingly and now only keep the same letter between specific blots and their quantification.

7. The P-values shown in figure 5J are difficult to parse.

© 2022. Published by The Company of Biologists under the terms of the Creative Commons Attribution License (https://creativecommons.org/licenses/by/4.0/).

We have tried to edit it accordingly and hope it is clear now.

8. It's not clear how the % pixel moving plots are measured or used.

We now cite our recent JCS paper from earlier this year that goes into greater details into the measurement, but it shows the number of moving pixels in the monolayer as a reflection of the percentage of cilia in the field.

9. It isn't always clear what the authors mean by cellular plasticity and the definition in the first line of the paper could be improved on.

We appreciate this and have tried to clarify this in our revision. In general, we define epithelial plasticity as cellular changes that alter its structure and shape with a resulting change in cellular function.

<u>Second decision letter</u>

MS ID#: JOCES/2021/258513

MS TITLE: Epithelial plasticity in COPD results in cellular unjamming due to an increase in polymerized actin

AUTHORS: Baishakhi Ghosh, Kristine Nishida, Lakshmana Chandrala, Saborny Mahmud, Shreeti Thapa, Carter Swaby, Si Chen, Atulya A Khosla, Joseph Katz, and Venkataramana Sidhaye
ARTICLE TYPE: Research Article

We have now reached a decision on the above manuscript.

To see the reviewers' reports and a copy of this decision letter, please go to: https://submit-jcs.biologists.org and click on the 'Manuscripts with Decisions' queue in the Author Area. (Corresponding author only has access to reviews.)

As you will see, two of the three reviewers continue to raise a number of substantial criticisms that prevent me from accepting the paper at this stage.

Referee #1 feels that the paper is still difficult to read. Importantly they also point out technical issues, questioning the effectiveness of knockdown and overexpression of Cofilin 1 (Figure 7), which raises concerns regarding the conclusion that cofilin is responsible for regulating the UJT or EMT. The differences shown may be just the variability across different cultures. They also suggest that apical cell shape be included as one of the metrics of the UJT. They would require that these issues be addressed in order to support publication.

Referee #3 points out that interpretation of your results rely heavily on precise values for the scaling exponents associated with the energy spectra, and you have not addressed the criticism that the data doesn't support such strong claims about the specific values of the exponents. I would refer you to their review where they elaborate on this point.

Normally with this level of remaining concerns I would not recommend we proceed with another revision. However, I would be amenable to reconsidering just one more revision, if you can convincingly address the referees' concerns. I would send the paper back out to referees 1 and 3.

We are aware that you may be experiencing disruption to the normal running of your lab that makes experimental revisions challenging. If it would be helpful, we encourage you to contact us to discuss your revision in greater detail. Please send us a point-by-point response indicating where you are able to address concerns raised (either experimentally or by changes to the text) and where you will not be able to do so within the normal timeframe of a revision. We will then provide further guidance. Please also note that we are happy to extend revision timeframes as necessary.

© 2022. Published by The Company of Biologists under the terms of the Creative Commons Attribution License (https://creativecommons.org/licenses/by/4.0/).

Please ensure that you clearly highlight all changes made in the revised manuscript. Please avoid using 'Tracked changes' in Word files as these are lost in PDF conversion.

I should be grateful if you would also provide a point-by-point response detailing how you have dealt with the points raised by the reviewers in the 'Response to Reviewers' box. Please attend to all of the reviewers' comments. If you do not agree with any of their criticisms or suggestions please explain clearly why this is so.

Reviewer 1

Advance summary and potential significance to field

The manuscript is improved greatly but remains difficult to read. Moreover, there are substantive issues that remain unresolved. The authors believe that CS exposure induces the Cofilin dependent state where the pEMT and UJT coexist. This may be possible but their data are unconvincing.

Comments for the author

Major:
The manuscript is improved greatly but remains difficult to read. Moreover there are substantive issues that remain unresolved. The authors believe that CS exposure induced the state where the pEMT and UJT coexist. It may be possible but their data are unconvincing.
1.The level of Cofilin 1 in the three lanes of Fig 7J look pretty similar, maybe a little less in KD, but overall not entirely convincing. This means that Cofilin 1 has not been knocked down in the KD(knockdown) or overexpressed in the OE (OE), as they claimed. This undermines their conclusion that cofilin is responsible for regulating the UJT or EMT. The differences shown may be just the variability across different cultures.
2. Could you show images of apical cell shape as one of the metrics of the UJT? You have the speed of cellular migration but do not include cell shape analysis. That would be the required data for the acceptance of the manuscript.
3. I recommend that you prepare the reader by saying something about the energy cascade analysis and its role in this manuscript somewhere in the introduction.
Minor:
CHBE is undefined 38 Either displayed, or display, not displays 46-49 These lines are unreadable/incomprehensible 58 adapt, not adapts 94 comparing, not compared
326 indicated, not indicted 415-415 “One could conclude that motility and local interactions do not seem to play a primary role in the present cultures.” Please explain your argument

Reviewer 2

Advance summary and potential significance to field

Manuscript message is much improved by removing data and clarifying message. It is of interest that cigarette smoke reduces cofilin levels and thereby impacts the cell jamming/unjamming state (via cofilin KD and OE experiments).

Comments for the author

No additions requested.

Reviewer 3

Advance summary and potential significance to field

As stated in the previous review, this paper makes an important advance and is high impact.

© 2022. Published by The Company of Biologists under the terms of the Creative Commons Attribution License (https://creativecommons.org/licenses/by/4.0/).

Comments for the author

The authors have responded to some of the comments by the reviewers, and performed additional analyses that strengthen their claims significantly. Nevertheless, there is still a major question about the precision of their exponents that should be addressed before publication.
The authors present new quantitative data that significantly strengthens their claim that there is an unjamming transition in some cell types. For example, they have added quantification of the cell displacements, which are consistent with the overall story: CHBE cells and those exposed to CS move more, while NHBE and cells that have undergone pEMT move less. They also demonstrate that cell shapes are more elongated in the layers that move more, consistent with previous work. In addition, they have made significant adjustments to the story to emphasize that they believe the turbulence metrics are important for distinguishing between and UJT and a pEMT. The new text explaining the results now makes it clear that characterizing the energy spectrum does distinguish between these different cell types. This is very nice. However, their interpretation of these results rely heavily on precise values for the scaling exponents associated with these energy spectra, and they have not addressed the criticism that their data doesn't support such strong claims about the specific values of the exponents.

Specifically, as the authors state in the rebuttal, it *is* clear that the exponent for the UJT systems is distinct from the exponent of roughly -4 associated with the Lin 2021 manuscript. On the other hand, it is absolutely not clear that they can distinguish a slope of -5/3 from -2 or -1.5. Therefore, it is also not clear that the mechanism driving the vortices here is energy cascades related to 2D turbulence. Their data is possibly consistent with such an interpretation, but currently their text makes it sound like their data confirms that this is definitely the mechanism. This is highly misleading. Given the data here, it seems reasonable to highlight that the energy spectrum can distinguish between cell types (which is indeed an interesting result) and that it is consistent with 2D turbulence in the UJT systems.

Second revision

<u>Author response to reviewers' comments</u>

Dear Green,

Thank you for taking our manuscript into consideration for publication in *Journal of Cell Science*, and the invitation to submit a revised manuscript for review. We want to thank the Reviewers for their time, thoughtful comments, and overall support for our manuscript. We are glad that they have found our revision improved, although Reviewers 1 and 3 do have some concerns, which we hope to address in this version. We have addressed each comment below, with our responses in blue.

Reviewer 1 Comments for the Author:

Major:

1. The manuscript is improved greatly but remains difficult to read.

We apologize for the remaining confusion and have tried to edit the text to improve readability.

2. Moreover, there are substantive issues that remain unresolved. The authors believe that CS exposure induced the state where the pEMT and UJT coexist. It may be possible but their data are unconvincing.

   a. The level of Cofilin 1 in the three lanes of Fig 7J look pretty similar, maybe a little less in KD, but overall not entirely convincing. This means that Cofilin 1 has not been knocked down in the KD(knockdown) or overexpressed in the OE (OE), as they claimed. This undermines their conclusion that cofilin is responsible for regulating the UJT or EMT. The

© 2022. Published by The Company of Biologists under the terms of the Creative Commons Attribution License (https://creativecommons.org/licenses/by/4.0/).

differences shown may be just the variability across different cultures.

We apologize for this. We have performed these experiments several times now, using samples derived from several different subjects and do identify this consistent finding. We have a different blot in our figure (Fig. 7J, L) and have included mRNA analyses (Fig. 7 K) supporting our findings.

b. Could you show images of apical cell shape as one of the metrics of the UJT? You have the speed of cellular migration but do not include cell shape analysis. That would be the required data for the acceptance of the manuscript.
We had included the cell shape analysis in our previous submission but hadn't included the images due to the bulkiness of the manuscript (Fig. 4K, Fig 7 T, U). We do include these images now. We see that with cofilin-1 knockdown the cells have an increase in the cell shape index, with evidence of longer cells. With cofilin- 1 overexpression, there is also an increase in the cell shape index, although the cells are much bigger, and are both longer and wider. However, the combination of cofilin-1 overexpression and CS, does cause the cells to be smaller with a cell shape index more similar to that of control cells (Fig. 7U).

c. I recommend that you prepare the reader by saying something about the energy cascade analysis and its role in this manuscript somewhere in the introduction.

We have added the following paragraph to the introduction of the revised manuscript: "Spectral analysis was used for characterizing and comparing the size, spatial distributions, and energy of cell motions within the different cultures. It enabled us to highlight similarities and discrepancies in the eddy sizes and strength among the different cases. Furthermore, the spectral slopes were compared to those of the kinetic energy of eddies and scalars in typical planar turbulent flows (e.g. Kraichnan, 1971, Sreenivasan, 2019; Gotoh et al., 2000), as well as to recently measured and simulated spectra of motile cell cultures (Lin et al., 2021). Since the present trends appeared to be more consistent with those of passive scalar motions, they suggested that motility and cell-cell interactions did not play primary roles in the cultures investigated here.

Minor:

CHBE is undefined

We would like to point the reviewer to line 37 in the abstract and lines 92-93 in the Introduction, where we defined CHBE. We have now underlined the first letters of COPD human bronchial epithelial in line 92 to highlight this abbreviation.

38 Either displayed, or display, not displays

We apologize for the error and have corrected these.

46-49 These lines are unreadable/incomprehensible

We apologize for the lack of clarity and have edited these lines accordingly.

58 adapt, not adapts, 94 comparing, not compared , 326 indicated, not indicted

Again, we apologize and have edited accordingly.

415-415 "One could conclude that motility and local interactions do not seem to play a primary role in the present cultures." Please explain your argument

Based on numerical simulations and measurements, Lin et al. (2021) have concluded that for cases involving cell motility and cell-cell interactions, the energy spectra of cell cultures have slopes

© 2022. Published by The Company of Biologists under the terms of the Creative Commons Attribution License (https://creativecommons.org/licenses/by/4.0/).

falling in the -4- to -4.5 range. In the present cultures, all the spetral slopes are considerably milder, around -5/3 at low wavenumbers and -1 at high wavenumber, trends that are more consistent with those of passive scalars in two dimensional turbulent flows (e.g. Kraichnan, 1971, Sreenivasan, 2019; Gotoh et al., 2000). These observations suggest that motility and cell-cell interactions do not play primary roles in driving the cell motions in the present cultures, as the authors conclusions suggest that to be the cause for the slope. We have revised the test to clarify this point. The revised version now reads (lines 412-421): "Recent studies involving cell-cell local interactions and cell motility, where the source of energy is at the scale of a pair of cells, found significantly steeper spectral slopes, -4.5 in experimental data and -4 in numerical simulations (Lin et al., 2021; Angelini et al., 2010). In our study the spectral slopes are considerably milder, about -5/3 at low wavenumbers and -1 at high wavenumbers. Such slopes are more consistent with those of the the classical motions of passive scalars in Kolmogorov-Kraichnan turbulence, with the transition occurring at the scale of about four cells (Kraichnan, 1971, Sreenivasan, 2019; Gotoh et al., 2000)). These trends suggest that motility and local interactions play less of a role in driving the motions of the present cultures since these interactions are thought to dictate these steeper slopes (Lin et al., 2021; Angelini et al., 2010).

Reviewer 3 Comments for the Author:

The authors have responded to some of the comments by the reviewers and performed additional analyses that strengthen their claims significantly. Nevertheless, there is still a major question about the precision of their exponents that should be addressed before publication.

Specifically, as the authors state in the rebuttal, it *is* clear that the exponent for the UJT systems is distinct from the exponent of roughly -4 associated with the Lin 2021 manuscript. On the other hand, it is absolutely not clear that they can distinguish a slope of -5/3 from -2 or -1.5. Therefore, it is also not clear that the mechanism driving the vortices here is energy cascades related to 2D turbulence. Their data is possibly consistent with such an interpretation, but currently their text makes it sound like their data confirms that this is definitely the mechanism. This is highly misleading. Given the data here, it seems reasonable to highlight that the energy spectrum can distinguish between cell types (which is indeed an interesting result) and that it is consistent with 2D turbulence in the UJT systems.

We agree the limited spectral range would make it difficult to distinguish between slopes that deviate slightly from -5/3, e.g. -2 or -1.5. However, these slopes are clearly very different from those modeled/simulated and measured by Lin et al. (2021) for motile cells. We have revised the text to indicate that the present trends are more consistent with those of passive scalars in 2D turbulence. The revised text (which is also included above) now states (lines 412-421):
"Recent studies involving cell-cell local interactions and cell motility, where the source of energy is at the scale of a pair of cells, found significantly steeper spectral slopes, -4.5 in experimental data and -4 in numerical simulations (Lin et al., 2021; Angelini et al., 2010). We do not see such steep trends in our study, where the spectral slopes are considerably milder, about -5/3 at low wavenumbers and -1 at high wavenumbers. Such slopes are more consistent with those of the the classical motions of passive scalars in Kolmogorov-Kraichnan turbulence, with the transition occurring at the scale of about four cells (Kraichnan, 1971, Sreenivasan, 2019; Gotoh et al., 2000)). These trends suggest that motility and local interactions do not play a primary roles in driving the motions of the present cultures since these interactions are thought to dictate these steeper slopes (Lin et al., 2021; Angelini et al., 2010)."

© 2022. Published by The Company of Biologists under the terms of the Creative Commons Attribution License (https://creativecommons.org/licenses/by/4.0/).

Third decision letter

MS ID#: JOCES/2021/258513

MS TITLE: Epithelial plasticity in COPD results in cellular unjamming due to an increase in polymerized actin

AUTHORS: Baishakhi Ghosh, Kristine Nishida, Lakshmana Chandrala, Saborny Mahmud, Shreeti Thapa, Carter Swaby, Si Chen, Atulya A Khosla, Joseph Katz, and Venkataramana Sidhaye
ARTICLE TYPE: Research Article

We have now reached a decision on the above manuscript.

To see the reviewers' reports and a copy of this decision letter, please go to: https://submit-jcs.biologists.org and click on the 'Manuscripts with Decisions' queue in the Author Area. (Corresponding author only has access to reviews.)

As you will see, referee #3 still raises some critical points that will need to be addressed regarding the interpretation of the exponents in the spectra and their connection to 2D turbulence, as well as the claim that this result implies something about reduced cell-cell interactions. The issues can be addressed by re-writing more extensively to accurately state what can be concluded from the data, and also I would ask that you quantify the uncertainty in the reported exponents, as the referee requests. Please explicitly state what wording is changed in your response.

We are aware that you may be experiencing disruption to the normal running of your lab that makes experimental revisions challenging. If it would be helpful, we encourage you to contact us to discuss your revision in greater detail. Please send us a point-by-point response indicating where you are able to address concerns raised (either experimentally or by changes to the text) and where you will not be able to do so within the normal timeframe of a revision. We will then provide further guidance. Please also note that we are happy to extend revision timeframes as necessary.

Please ensure that you clearly highlight all changes made in the revised manuscript. Please avoid using 'Tracked changes' in Word files as these are lost in PDF conversion.

I should be grateful if you would also provide a point-by-point response detailing how you have dealt with the points raised by the reviewers in the 'Response to Reviewers' box. Please attend to all of the reviewers' comments. If you do not agree with any of their criticisms or suggestions please explain clearly why this is so.

Reviewer 1

Advance summary and potential significance to field

My previous concerns have been addressed. This is a good contribution to the archive because it links pathology, cell biology, signaling and sophisticated physics in a very novel way. I support publication.

Comments for the author

My previous concerns have been addressed. This is a good contribution to the archive because it links pathology, cell biology, signaling and sophisticated physics in a very novel way. I support publication.

© 2022. Published by The Company of Biologists under the terms of the Creative Commons Attribution License (https://creativecommons.org/licenses/by/4.0/).

Reviewer 3

Advance summary and potential significance to field

Same as the last two reviews I provided.

Comments for the author

This is now the 3rd version of this manuscript I have seen. I think that both the first reviewer and I have a very similar set of concerns about the interpretation of the exponents in the spectra and their connection to 2D turbulence, as well as the claim that this result implies something about reduced cell-cell interactions. The authors have made small changes to one descriptive part of text in response to these concerns, although a major section of their results still contains strong language connecting their observations to passive 2D turbulence. In addition, they have not quantified the uncertainty in their reported exponents, as requested.

My feeling is that there is a lot of good work in this manuscript, but the authors have essentially refused to address a significant concern about interpretation of their results raised by multiple referees. At the end of the day, the editor has to consider the full balance of the value of the article and decide whether that's okay or not.

Third revision

Author response to reviewers' comments

Dear Green,
Thank you for taking our manuscript into consideration for publication in the *Journal of Cell Science*, and for the invitation to submit a revised manuscript for review. We are glad that with our revision, Reviewer 1 has no further concerns with our interpretation and conclusion and supports this publication. Reviewer 3 has some remaining concerns which we will try and address in this version, with our responses in blue.

Reviewer 3 Comments for the Author:
This is now the 3rd version of this manuscript I have seen. I think that both the first reviewer and I have a very similar set of concerns about the interpretation of the exponents in the spectra and their connection to 2D turbulence, as well as the claim that this result implies something about reduced cell-cell interactions.

While we apologize for not addressing Reviewer 3's concerns adequately, we would like to point out that the first reviewer has not raised any further concerns and is satisfied with both our interpretation and the claims we state. Perhaps, we do not fully understand what Reviewer 3 is trying to raise. Our comments about cell-cell interactions are in response to a recently published paper (Lin et al., 2021), which was raised by Reviewer 1 in the previous review in which we were asked to address the differences. Our data show a much milder slope than those modeled/simulated and measured by Lin et al. (2021) for motile cells. Their spectral slopes range between -4 to -4.5, and ours (Figure 8) range from about -5/3 at low wavenumbers, to -1 at high wavenumbers. To highlight the difference between slopes, in the present version, we have added a line segment with a -4 slope (along with the -5/3 and-1 slope line) in the bottom left corner of each plot. Clearly, irrespective of the assessed uncertainty, our spectra are clearly significantly milder than those in Lin et al. Based on their numerical simulations, which included terms involving motility and cell-cell local interactions, Lin et al. argued that their steep slopes were caused by the motility and local interactions. Considering that our present slopes are significantly milder, and show trends that are more consistent with those of *passive* 2D turbulent flows (e.g. -5/3 in the inertial range), we postulate that in our cultures motility and local interactions do not play as a significant role as those in Lin et al. This comment is made in the line 405.

© 2022. Published by The Company of Biologists under the terms of the Creative Commons Attribution License (https://creativecommons.org/licenses/by/4.0/).

The authors have made small changes to one descriptive part of text in response to these concerns, although a major section of their results still contains strong language connecting their observations to passive 2D turbulence. In addition, they have not quantified the uncertainty in their reported exponents, as requested.

We state in lines 304-306 that we are interpreting the data based on the model of 2D turbulence in a specific range. We have highlighted this in the manuscript. Our so-called "strong language" simply states that we make an analogy to 2D turbulence. In response to the original criticisms that were raised by the reviewer, we added error bars to the spectra and outlined the methodology for the generation of these bars (bootstrap method, commonly used in field environmental data), as we thought this would quantify the uncertainty in the data. Now, we also have also listed the uncertainty in the slope as generated by these error bars. By fitting lines with different slopes through the range covering the error bars, our slopes deviate from -5/3 by significantly less than +-0.33, and we have included this in the manuscript (line 303). As noted above, we have also added a line with a -4 slope to highlight how different our trends are from those predicted by the Lin et al. model and measurements to occur in cases where motility and local interactions play a significant role.

Fourth decision letter

MS ID#: JOCES/2021/258513

MS TITLE: Epithelial plasticity in COPD results in cellular unjamming due to an increase in polymerized actin

AUTHORS: Baishakhi Ghosh, Kristine Nishida, Lakshmana Chandrala, Saborny Mahmud, Shreeti Thapa, Carter Swaby, Si Chen, Atulya A Khosla, Joseph Katz, and Venkataramana Sidhaye
ARTICLE TYPE: Research Article

I am happy to tell you that your manuscript has been accepted for publication in Journal of Cell Science, pending standard ethics checks.

© 2022. Published by The Company of Biologists under the terms of the Creative Commons Attribution License (https://creativecommons.org/licenses/by/4.0/).
